# Developing and Characterizing a Biocompatible Hydrogel Obtained by Cross-Linking Gelatin with Oxidized Sodium Alginate for Potential Biomedical Applications

**DOI:** 10.3390/polym16223143

**Published:** 2024-11-11

**Authors:** Chahrazed Mahmoudi, Naïma Tahraoui Douma, Hacene Mahmoudi, Camelia Elena Iurciuc (Tincu), Marcel Popa, Mihaela Hamcerencu, Călin Vasile Andrițoiu

**Affiliations:** 1 Laboratory of Water and Environment, Faculty of Technology, University Hassiba Benbouali of Chlef, Chlef 02000, Algeria; c.mahmoudi@univ-chlef.dz (C.M.); n.tahraoui@univ-chlef.dz (N.T.D.); 2 Department of Natural and Synthetic Polymers, Faculty of Chemical Engineering and Protection of the Environment, “Gheorghe Asachi” Technical University, 700050 Iasi, Romania; 3 National Higher School of Nanosciences and Nanotechnologies, Algiers 16000, Algeria; hac.mahmoudi@gmail.com; 4 Department of Pharmaceutical Technology, Faculty of Pharmacy, “Grigore T. Popa” University of Medicine and Pharmacy, University Street, No. 16, 700115 Iasi, Romania; 5 Academy of Romanian Scientists, 3 Ilfov, 050044 Bucharest, Romania; 6 CQFD Composites, 2 Rue du Maine, 68270 Wittenheim, France; mhamcerencu@yahoo.com; 7 Department of Nutrition and Dietetics, Faculty of Pharmacy, Vasile Goldis Western University of Arad, Liviu Rebreanu Street, 86, 310045 Arad, Romania; dr_calin_andritoiu@yahoo.com

**Keywords:** oxidized sodium alginate, hydrogel, gelatin, propolis immobilization, delivery system

## Abstract

The main goal of this research was to create biocompatible hydrogels using gelatin and a double cross-linking technique involving both covalent and ionic bonds to immobilize propolis. The covalent bonds were formed through Schiff base cross-links between protein-free amino groups (NH_2_) from the lysine residue and aldehyde groups (CHO) produced by oxidizing sodium alginate with NaIO_4_, while the ionic bonds were achieved using Mg^2+^ ions. Hydrogel films were obtained by varying the molar ratios of –CHO/–NH_2_ under different pH conditions (3.5 and 5.5). The presence of aldehyde groups in the oxidized sodium alginate (OSA) was confirmed using FTIR and NMR spectroscopy. The oxidation degree was monitored over 48 h, and the influence of temperature was examined. Results showed that higher –CHO/–NH_2_ molar ratios led to increased conversion index values of NH_2_ groups, and a decrease in swelling degree values was observed in mediums with pH values of 5.5 and 7.4. The encapsulation and release efficiency of propolis decreased with an increase in the hydrogel cross-linking degree. UV irradiation enhanced the antioxidant activity of both free and encapsulated propolis. These findings offer valuable insights for the biomedical and pharmaceutical fields into designing biocompatible hydrogels for propolis immobilization, with potential for controlled release.

## 1. Introduction

The successful delivery of drugs is a complex process that involves the integration of several disciplines, such as chemistry, pharmaceutical sciences, medicine, and engineering, and relies heavily on the chemical formulation of drugs, the type of dosage form, and the route of administration [1,2,3]. Drug delivery systems aim to overcome the limitations of conventional drug administration methods by enhancing drug solubility, prolonging the duration of drug action, reducing side effects, and preserving drug bioactivity [4]. However, despite advances in drug delivery technology, the limited effectiveness of many active ingredients remains a significant challenge. Often, these active ingredients are degraded before reaching their intended target, or they are administered at suboptimal doses, which fail to meet the patient’s therapeutic needs. Biopolymer systems that provide controlled local delivery of active ingredients appear an attractive solution to overcome these limitations, allowing spatial and temporal control of the release of the encapsulated molecule.

Recently, the combination of biopolymers has provided an ideal platform for delivering therapeutic agents, such as drugs and proteins [5,6]. Hydrogels based on biopolymers are an essential class of materials that has attracted considerable attention in recent years, particularly for drug delivery applications [7]. By definition, hydrogels are three-dimensional networks of hydrophilic polymers that can absorb and retain large amounts of water. They have been extensively studied for drug delivery due to their ability to protect drugs from degradation, control their release rate, and target specific tissues or cells [5,7]. Biopolymer-based hydrogels offer additional advantages such as biocompatibility, biodegradability, and the ability to interact with biological molecules [8,9].

In order to create an effective controlled drug delivery system using biopolymers, the primary phase is selecting the appropriate biopolymer(s). This requires thoroughly comprehending biopolymers’ surface and intrinsic characteristics, which are critical for designing drug delivery systems to achieve maximum therapeutic benefits [5,10]. The choice of biopolymer for hydrogel synthesis can affect the properties of the resulting hydrogel, including its mechanical strength, porosity, and responsiveness to external stimuli. Some commonly used biopolymers for hydrogel synthesis include alginate, chitosan, hyaluronic acid, collagen, and gelatin.

Gelatin (Gel), a derivative of collagen obtained by its partial hydrolysis, is a water-soluble compound that readily dissolves at 37 °C, does not trigger an immune response, and displays amphoteric behavior [11,12,13]. Gel’s molecular weight and properties are influenced by two primary factors: the collagen source and the manufacturing process. Specifically, the type of collagen used and the method of manufacturing (e.g., heat and enzymatic denaturation or extraction in either alkaline conditions, which forms gelatin type B, or in acidic conditions, which forms gelatin type A) can have a significant impact on the resulting Gel’s molecular weight, viscosity, and gelling strength [14]. The structural features of Gel include the presence of a repeating amino acid sequence and a high concentration of hydroxyproline and hydroxylysine. As a result of these unique properties, hydrogels made from Gel are widely utilized in developing delivery systems for drugs, and are also used as composite for contact lenses and matrices for tissue engineering [14].

Furthermore, the mechanical and chemical characteristics of Gel can be modulated through the use of different cross-linking agents, for example, glutaraldehyde [15], genipin [16], and dialdehyde from oxidized polysaccharides [17,18]. Sodium alginate (SA) is a non-toxic and naturally occurring polysaccharide extracted from brown seaweed, such as kelp, which is widely used in the food [19], pharmaceutical [20], and textile industries [21]. It is a water-soluble salt that forms a viscous, gel-like substance when it comes into contact with water, making it an ideal thickener, stabilizer, and emulsifier. SA can be modified by oxidation using sodium periodate in an aqueous solution, decreasing its molecular weight and improving its reactivity and biodegradability [22,23]. This modification allows for a wider range of applications for SA in industries such as food, pharmaceuticals, and biomedicine. The oxidation modification of SA involves the hydroxyl groups at the C-2 and C-3 vicinal bonds in the uronic units of the alginate chains being transformed into dialdehyde groups [24]. These dialdehyde groups can then undergo further chemical reactions, such as cross-linking, to form more complex structures with different properties. Oxidized sodium alginate (OSA)-based hydrogels have shown promise in a variety of applications, including drug delivery [25], tissue engineering [26], and wound healing [27]. Carbonyl groups in the hydrogel can react with various functional groups in drugs or proteins, allowing the controlled release of these molecules over time, while the reaction of amino groups can lead to hydrogels with enhanced cell adhesion properties, making them ideal for use in several applications [28]. Furthermore, the varying cross-linking degree of OSA-based hydrogels makes them helpful in creating scaffolds for tissue engineering and drug delivery systems with improved stability and durability [29].

The Gel-OSA-based hydrogel stands out as one of the most abundant systems, possessing several advantageous properties for developing functional materials, such as biocompatibility, nontoxicity, flexibility, and biodegradability [24,30]. When the free amino groups of lysine and hydroxylysine residues in Gel react with the aldehyde groups of OSA, covalent bonds are formed through Schiff base linkages. This reaction produces highly stable and physiologically degradable networks, which exhibit improved properties of cell adhesion and biocompatibility. Pettignano et al. [30] synthesized a Gel-based hydrogel cross-linked with OSA using borax to yield hybrid hydrogels with self-healing properties due to reversible Schiff base formation, resulting in a bio-hydrogel that combines the properties of both materials and has the unique capability to self-repair when subjected to mechanical damage. Their results suggested that achieving the best self-healing properties for these hydrogels requires a careful balance between concentration, the stoichiometric ratio of the two biopolymers, and the source type of Gel. Moreover, the healing process was found to be significantly influenced by the pH, indicating that the dynamic Schiff base linkages between the amine groups of Gel type B and the aldehyde groups of OSA play a crucial role in the reconstruction of the damaged hydrogel interface [30].

According to a study conducted by Rottensteiner et al. [31], alginate–gelatin hydrogels displayed favorable in vitro and in vivo biocompatibility and exhibited strong cell adhesion and proliferation of mesenchymal stem cells. These promising findings suggest that alginate–gelatin hydrogels could be valuable in bone tissue engineering and warrant further investigation in this area [31].

Propolis (Pro) is a natural, resinous substance that is collected and processed by bees from various plant sources [32]. The content of propolis consists of over 300 components [33]. It is high in resin, gummy substances, waxes, essential oils, aromatic oils, phenolic components, and bioactive compounds (such as phenolic acids, flavonoids, ketones, and aldehydes) [33,34]. Generally, Pro compositions depend on the botanical origin [35]. Pro’s abundance of chemical compounds contributes to its various biological activities, which vary among different geographical samples. These activities include anesthetic, antimicrobial, antioxidant, anti-inflammatory, and, more recently, antiproliferative and antitumor effects [34,36]. Furthermore, Pro is non-toxic, safe, and capable of exhibiting antimicrobial synergism when administered in combination with certain antibiotic drugs [37].

Recent advancements in the incorporation of propolis into biopolymer hydrogels have opened new possibilities for the development of biomaterials with enhanced antimicrobial and antioxidant properties. These hydrogels, often composed of natural polymers such as chitosan, alginate, or gelatin, provide a promising platform for biomedical applications, including wound dressings, drug delivery systems, and tissue engineering scaffolds [38,39,40].

However, despite the potential benefits of biopolymer hydrogels, these systems face significant limitations that hinder their long-term effectiveness. One of the primary challenges is the rapid release of propolis from the hydrogel matrix, which often occurs within the first few hours of application. This burst release can result in a short-lived therapeutic effect, reducing the efficacy of the propolis over extended periods. Moreover, biopolymer hydrogels are known for their high swelling degree, especially within the first hour of application, which can lead to structural instability and uncontrolled release kinetics, as demonstrated by Pangesty et al. [41]. and Phonrachom et al. [42].

In contrast, the composition developed in this study addresses these limitations by providing a controlled and sustained release of propolis’s active components. Through carefully selecting polymeric materials and optimizing film-forming techniques, our formulation allows for a gradual diffusion of the bioactive compounds, ensuring prolonged antimicrobial and antioxidant effects. Additionally, the swelling degree of our films is regulated to maintain structural integrity, further enhancing their potential for long-term applications, making the hydrogel films more effective over time, and offering superior performance compared to hydrogel-based systems, particularly in extended-release applications where consistent therapeutic effects are required.

This study’s primary goal and novel contribution was to optimize the reaction parameters for producing hydrogel films using Gel cross-linked with OSA to achieve the most favorable physicochemical properties for the controlled and sustained release of Pro, including longer release time and higher release efficiency. Three phenolic acids, caffeic acid, p-coumaric acid, and ferulic acid, were quantified using Pro [43]. Para-coumaric acid was used as Pro’s essential component to study the prepared hydrogels’ encapsulation and release performances.

The obtained OSA structure was analyzed using FTIR and ^1^H-NMR spectroscopy. The oxidation degree was determined, and the effects of varying oxidation times and temperatures were assessed. We also examined the ability of aldehyde groups within OSA to cross-link the free amino groups within gelatin by analyzing the conversion index of NH_2_ groups to Schiff bases using different NH_2_/CHO molar ratios and two different pH mediums. The hydrogels obtained with and without encapsulated propolis were characterized using FTIR spectroscopy, scanning electron microscopy, and swelling degree. Additionally, we tested the protective role of the polymer matrix by evaluating the antioxidant activity of propolis-containing films exposed to UV light for 30 min. Furthermore, we assessed the ability of the hydrogel films to control and sustain the release of p-coumaric acid within propolis in vitro in buffer solutions at pH = 5.5 and pH = 7.4.

## 2. Materials and Methods

### 2.1. Materials

Medium molecular weight Gel type A and native sodium alginate (SA) were purchased from Sigma Aldrich (St. Louis, MO, USA); Pro solution (30%) was a gift from Apitherapy Medical Center, Balanesti Romania; Tween 80, p-coumaric acid (PCA), sodium meta periodate, ninhydrin, glutamic acid, and dialysis membranes with pores of 14 kDa were purchased from Sigma Aldrich; sodium thiosulfate, acetic acid, potassium iodide, sodium chloride, disodium phosphate, monosodium phosphate, ethanol, and sodium hydroxide (NaOH) were purchased from Chemical Company, Iași, Romania.

### 2.2. Preparation of Oxidized Sodium Alginate (OSA)

**Figure 1:**
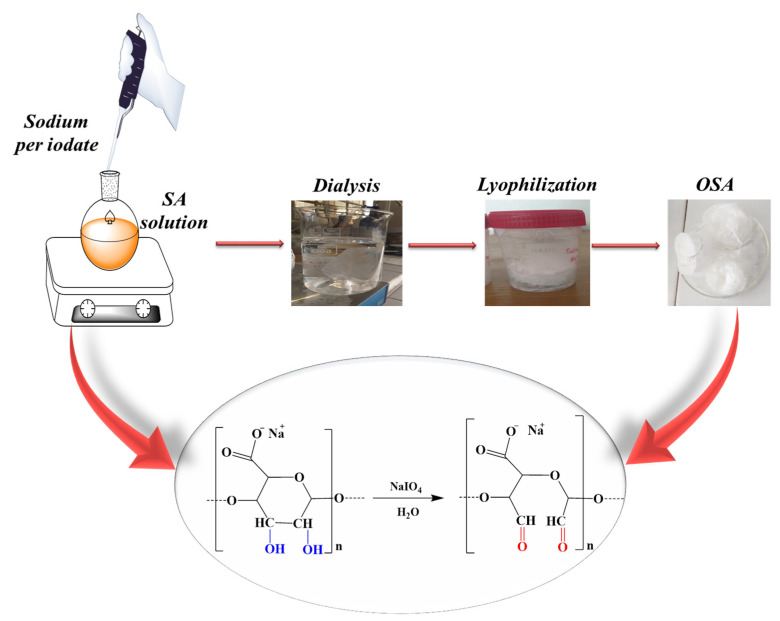
Schematic illustration of the experimental workflow and protocol for the preparation of oxidized sodium alginate (OSA).

The OSA was prepared using NaIO_4_ as an oxidizing agent for SA in aqueous solution (Figure 1) as follows: 2 g SA was solubilized in 100 mL of bi-distilled water at 80 °C. The resulting solution was then transferred into a 250 mL flask and left to cool down to 30 °C. A calculated stoichiometric amount of 1.5 g NaIO_4_ was dissolved in 20 mL of bi-distilled water at room temperature and added dropwise over the SA solution at 30 °C. The oxidation reaction took place in the dark for 24 h. The resulting product was purified by dialysis against deionized ultra-pure water for 3 days (the distilled water was changed at least five times) and finally lyophilized. The oxidized product was preserved in a desiccator at room temperature until the subsequent use.

### 2.3. Preparation of Hydrogel Films Based on Gelatin and OSA

The Gel/OSA-based hydrogels were prepared by mixing OSA and gelatin with different –CHO/–NH_2_ molar ratios (0.5:1, 0.75:1, 1:1, 1.5:1 mol/mol) in a 0.1 M acetate buffer solution (ABS) at two different pH levels (pH = 5.5 and 3.5; Table 1) in order to control and compare the hydrogel formation process in different acidic conditions. The number of moles of –NH_2_ groups from Gel was the same in all experiments (3.38 moles/0.2 g Gel), and the number of moles of OSA varied.

**Figure 2:**
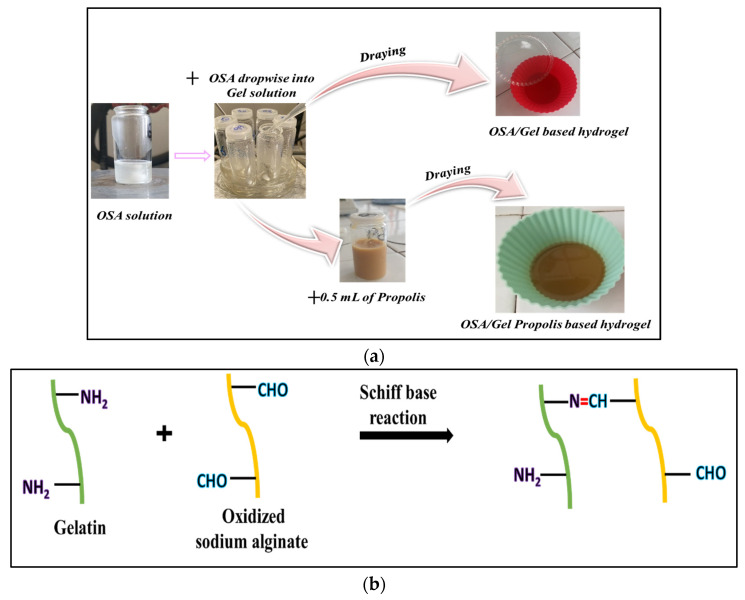
Schematic illustration of (**a**) experimental work of Gel/OSA-based hydrogel preparation and (**b**) reaction of Schiff base formation between –NH_2_ of gelatin and –CHO of OSA.

Figure 2 represents the experimental steps of the hydrogel’s preparation. First, the required amount of OSA was dissolved in ABS solution at 80 °C for 30 min. After cooling the OSA solution at 40 °C, 1.2 mL of Mg^2+^ aqueous solution (5% *w*/*v*) was added to the oxidized sodium alginate solution to react with its carboxyl groups (COO–) [44]; the mixture was placed in an ultrasonic bath for 15 min. The prepared solution was then added dropwise over the gelatin solution (0.2 g of gelatin dissolved in 2 mL of ABS at 40 °C) and maintained under stirring for 6 h. In order to obtain flexible, fragile-free hydrogels that could be applied to the skin, 1% glycerine *g*/*g* was added to all formulations. In order to obtain the hydrogel films, the biopolymer mixture at 40 °C was poured into silicon forms with a diameter of 4.5 cm and dried at room temperature. Once dried, the films were carefully removed from the silicon forms and stored in a refrigerator at 4 °C until further analysis.

The hydrogels containing Pro were prepared using the same protocol described previously. After stirring the biopolymer solution for 6 h to allow the cross-linking reaction to occur, 0.5 mL of 30% propolis hydroalcoholic solution was added to the biopolymer mixture. The mixture was stirred for 2 min and then sonicated in an ultrasonic bath at 40 °C for 30 min.

The experimental program is given in Table 1, and Figure 2 schematically shows the working method for obtaining hydrogel.

### 2.4. Characterization Methods

#### 2.4.1. Fourier-Transform Infrared Spectroscopy

The FTIR analysis was conducted to identify the chemical structure of SA, OSA, and Gel/SA, Gel/OSA, and Gel/OSA/propolis-based hydrogels. The spectra were recorded on a Bruker Vertex FTIR (Karlsruhe, Germany) spectrophotometer. The samples were pressed as KBr pellets and analyses were performed over the wavenumbers ranging from 400–4000 cm^−1^, at room temperature, and using a resolution of 4 cm^−1^.

#### 2.4.2. Nuclear Magnetic Resonance Spectroscopy of SA and OSA

^1^H NMR (Bruker NEO 1-400, Billerica, MA, USA) spectra were performed to establish the molecular structure of the sodium alginate before and after oxidation. The samples (SA, OSA) were dissolved in deuterium oxide, and then ^1^H NMR was used to analyze them.

#### 2.4.3. Quantification of Aldehyde Groups in OSA

In order to determine the aldehyde group content in the resulting OSA, a modified protocol of Dellali et al. [44] was carried out. The amount of aldehyde group present in OSA was indirectly measured by dosing residual sodium periodate by iodometric titration, and Lange previously described the reactions involved in 1961 [45]. Briefly, in a beaker, a 1 mL volume of the OSA solution from the oxidation reaction mixture was added to 1 mL of 20% KI solution mixed with 1 mL of 37% HCl. The formed I_2_ was titrated with 0.05 N Na_2_S_2_O_3_ until the endpoint, in which the solution color turned blue and then became transparent, which indicated the disappearance of I_2_ visualized by using 1 mL of 1% starch solution. Due to the titration reaction, the unreacted amount of sodium periodate in the oxidation reaction was determined by subtracting the amount of NaIO_4_ that was non-reacted from the initial amount of NaIO_4_ added. In order to calculate the number of moles of aldehyde groups produced in the reaction, we considered the reaction’s stoichiometry: it takes 1 mole of NaIO_4_ to produce 2 moles of aldehyde groups in the OSA.

#### 2.4.4. Oxidation Kinetics and Temperature Effect Studies

The study of the kinetics of the oxidation reaction experiment was performed at 30 °C, where 100 mL of an aqueous solution of SA (2% g/dL) was oxidized using a stoichiometrically calculated NaIO_4_ amount with an excess of 34% in the dark in a flask covered with aluminum folium. At each hour for up to 72 h, 1 mL of the reaction mixture was collected and titrated with Na_2_S_2_O_3_ to monitor the progress of the oxidation reaction. The degree of oxidation (OD%) was calculated using Equation (1).

(1) $$OD\%=\frac{M_{R}}{M_{T}}$$

M_R_ represents the amount of NaIO_4_ that reacted at each interval, and M_T_ represents the total NaIO_4_ amount calculated stoichiometrically that was added to the reaction.

In order to study the influence of the temperature on the performance of the oxidation reaction, we followed the same work protocol used in the oxidation kinetic studies described previously. The samples were prepared at different temperatures ranging from 25 to 45 °C for 24 h. Afterward, we collected 1 mL of the reaction mixture from each sample and titrated it with Na_2_S_2_O_3_.

#### 2.4.5. Thermogravimetric Analysis (TGA)

Thermogravimetric analysis (TGA) of SA, OSA, Gel, and Pro and the hydrogels SA/Gel, OSA/Gel, and OSA/Gel/Pro were analyzed using the TA instrument, model TGA Q500. The experiments were conducted under atmospheric air with a dynamic heating rate of 10 °C min^−1^. The temperature range for the analyses was set from 20 to 700 °C.

#### 2.4.6. Scanning Electron Microscopy

The surface morphology of hydrogel films was analyzed through scanning electron microscopy (SEM) after drying and gold metallization using a spray deposition device. The analysis was conducted with a JCM-6000 scanning electron microscope (Mitaka, Tokyo, Japan) using an accelerating voltage of 15 Kv.

#### 2.4.7. The Determination of Amino Groups’ Conversion Index (CI%) into Schiff Bases in Hydrogel Films

The amino group concentration within the Gel that reacts with the aldehyde groups within OSA in the cross-linking reaction to form Schiff bases was evaluated using the ninhydrin test. A stock solution of 0.1% (*w*/*v*) of glutamic acid in ABS, pH = 5.6, 0.1 M was prepared to plot the calibration curve. Different volumes were taken from the stock solution to prepare six different glutamic acid concentrations between 0.04 and 0.1 mg/mL. In order to plot the calibration curve, the following ninhydrin test protocol was employed: 1 mL of glutamic acid solution with varying concentrations, which had been previously prepared, was mixed with 2 mL of a 2% ninhydrin solution in ethanol in a test tube. The resulting mixture was then heated at a temperature of 95 °C for 30 min, causing the solution to turn dark blue. Once the solution had cooled, a mixture of 8 mL of ethanol and distilled water in a 1:1 volume ratio was added. In order to calibrate the spectrophotometer, a blank solution was prepared without glutamic acid, as previously mentioned. In place of glutamic acid, 1 mL of acetate buffer with a pH of 5.6 was added to the blank solution for calibration. The UV spectrophotometer was utilized to record the absorbances of the solutions at a wavelength of 570 nm. The initial concentrations of the solutions, measured in mg/mL, were then converted into molar concentrations, denoting the number of moles of amino groups per milliliter [46].

The ninhydrin test was necessary to determine the quantity of unbounded amino groups in the obtained films. A small fragment of the films was weighed and then added to test tubes, followed by the addition of 1 mL of 0.1 M acetate buffer with pH = 5.6 and 2 mL of 2% ninhydrin solution. The resulting mixture was kept for one hour at room temperature to allow the ninhydrin solution to diffuse into the films and then heated at 95 °C for 30 min. An 8 mL mixture of ethanol and bi-distilled water in a 1:1 volume ratio was added after cooling the solution. The absorbance of the resulting solution was measured at a wavelength of 570 nm. The quantity of unbound amino groups in the hydrogel films was determined using the glutamic acid calibration curve established earlier [47]. This enabled the calculation of the total number of amino groups that participated in the formation of Schiff bases using the difference between the total number of moles of free amino groups initially added in the reaction (before cross-linking) and the number of moles of free amino groups present after the reaction (after the cross-linking). The Equation from the calibration curve for glutamic acid was y = 0.117x. The conversion index was determined using Equation (2):

(2) $$CI\left(\%\right)=\frac{N_{a}-N_{b}}{N_{a}}\times 1$$

where N_a_ refers to the number of moles of free amino groups present before cross-linking, while N_b_ represents the number of moles of free amino groups present after cross-linking. The difference between N_a_ and N_b_ represents the number of amino groups that have formed covalent bonds (Schiff base) within the hydrogel film.

#### 2.4.8. Swelling Behavior of OSA/Gel Based Hydrogels

In order to determine the capacity of the OSA/Gel hydrogels to absorb and retain aqueous medium without dissolution, a swelling degree experiment was carried out. This characteristic is significant as it leads to the varying levels of diffusion of the active ingredient from the hydrogel matrix.

A traditional gravimetric method in buffer solutions was utilized to examine the swelling behavior of samples. A phosphate buffer at pH = 7.4 simulated physiological blood pH. An acetate buffer at pH = 5.5 was employed for skin pH simulation. The detailed procedure involved accurately weighing amounts of dried hydrogels (W_dry_) immersed in 5 mL of solution of different pH values at 37 °C. The samples were periodically taken out of the liquid, the excess water was removed from the surface using filter paper, and the swollen films were weighed (W_swelled films_). After weighing, the films were placed back into the solution.

This process was repeated at regular time intervals until the system reached equilibrium. The difference between the swollen film’s weight (W_swelled films_) and the weight of the dry film (W_dry_) represented the aqueous solution absorbed by the hydrogels (W_solution_). The degree of swelling was determined by calculating the ratio of the amount of solution retained by the films at each time interval to the weight of the completely dry film (Equation (3)).

(3) $$Q(\%)=\frac{W_{solution}}{W_{dry}}\times 100$$

#### 2.4.9. Encapsulation Efficiency

Due to propolis’s complex composition, determining its encapsulation and release is practically challenging. Nevertheless, researchers have evaluated the quantity of para-coumaric acid (PCA), the main component in Pros’ composition. The 500 μL Pro liquid used in this study contained 22.75 mg of PCA, as determined by HPLC [43].

To immobilize the Pro in the hydrogel matrix, the liquid Pro was added to the resulting polymer solution, and the whole system was put in an ultrasonic bath at 37 °C for 30 min. After that, the solution was placed in Petri dishes for 48 h to dry.

**Figure 3:**
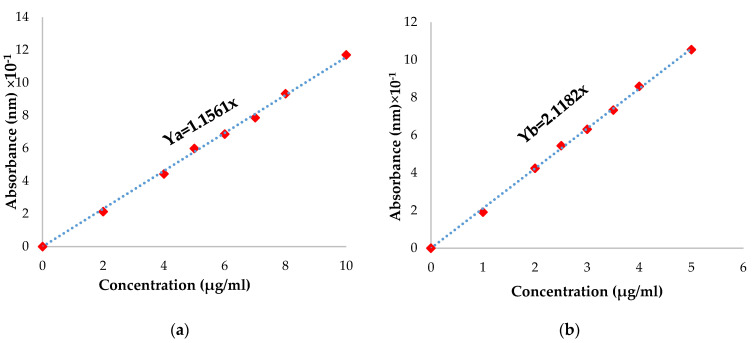

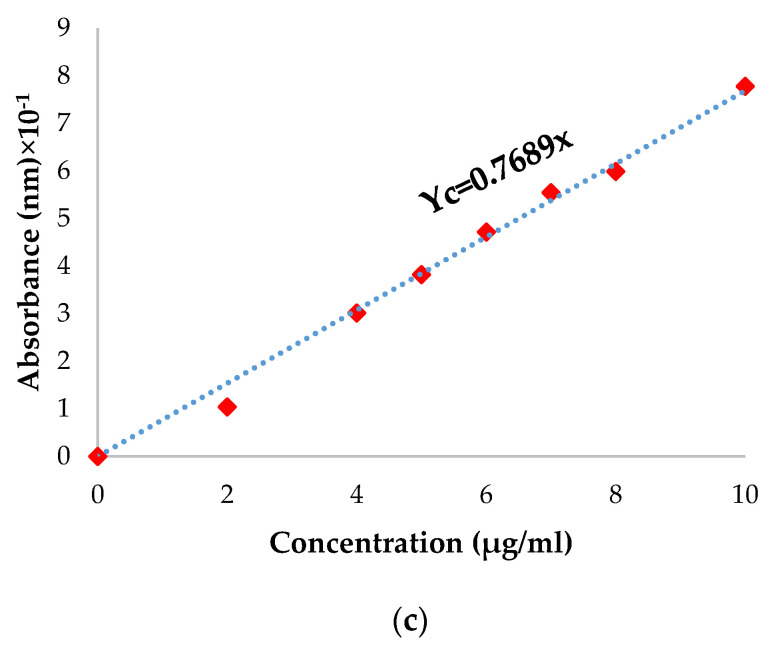
Calibration curve of PCA in: (**a**) pH 7.4 with 1% Tween 80 (*w*/*w*); (**b**) pH 5.5 with 1% Tween 80 (*w*/*w*); (**c**) ethanol.

The Pro quantity that was not immobilized was determined by dosing the para-coumaric acid. A PCA calibration curve was plotted (Figure 3) using the following procedure: 5 mg of PCA was dissolved in 10 mL solution containing 1% Tween 80 solution. The mixture was poured into a flask with 50 mL ABS (pH = 5.5) or PBS (pH = 7.4) solution containing 1% Tween 80. Seven different concentrations of PCA ranging from 0.01 to 0.05 mg/mL were prepared using varying volumes. The absorbance of the solutions was measured using a UV spectrophotometer at wavelengths of 299 nm and 287 nm for ABS (pH = 5.5) and PBS (pH = 7.4), respectively. The calibration curve displayed absorbance as a function of the concentration of PCA.

In order to determine the p-coumaric acid quantity from propolis that was immobilized, a fragment of each film was weighed and immersed in 1 mL of bi-distilled water for one hour; after that, 10 mL of ethanol was added and stirred for 24 h at 37 °C. The quantity of p-coumaric acid in each film was determined using the equation derived from the calibration curve of p-coumaric acid in ethanol (Figure 3). The encapsulation efficiency was determined with Equation (4):

Ef% = (Pro amount encapsulated)/(initial Pro amount) × 100 (4)

#### 2.4.10. Release Efficiency

To determine the release kinetics of Pro immobilized into the hydrogel matrix, two different pH media were used: 0.1 M acetate buffer solution at pH = 5.5 (similar to skin pH) and 0.1 M phosphate buffer solution at pH = 7.4 (similar to blood pH), at 37 °C, until equilibrium.

A fragment of the dried film was immersed in bakers containing 20 mL of ABS (pH = 5.5) and PBS (pH = 7.4) solution. Periodically, a sample of 0.250 mL was taken, and 2.75 mL of buffer solution was added to maintain equilibrium. The Pro amount released was determined spectrophotometrically based on the previously plotted PCA calibration curves in buffer solutions.

#### 2.4.11. Antioxidant Activity

The protocol method, initially outlined by Choi et al. [48], was employed with certain modifications in this study. Six samples of OSA/Gel-based hydrogels were utilized, each encapsulating Pro for subsequent antioxidant activity assessment. The hydrogels were divided into two groups. Pro extraction was performed in the first set by immersing half of the hydrogels directly in 20 mL of ethanol for 24 h. The second set of hydrogels was subjected to UV irradiation for 30 min before undergoing the same 24 h ethanol immersion.

In parallel, 0.250 mL of free Pro underwent UV irradiation for 30 min, followed by immersion in 20 mL of ethanol. Additionally, an equivalent volume of Pro was dissolved in 20 mL of ethanol without UV irradiation. Subsequently, each resulting sample served as a stock solution. In order to assess antioxidant activity, several dilutions were prepared from the stock solutions. The final concentrations of the Pro solutions ranged from 5 to 25 µg/Ml. In a set of test tubes, 2 mL of each solution concentration was introduced, followed by 2 mL of a 0.1 mM DPPH solution (in ethanol). After vortexing the samples for 20–30 s, they were placed in the dark at 37 °C for 1 h. Subsequently, the absorbance of the samples was measured using a UV spectrophotometer at a wavelength of 517 nm. Ascorbic acid was used as the standard. The resulting absorbance values were then transformed into percentages representing antioxidant activity (free radicals inhibition percentage in DPPH-I%) using Equation (5):

(5) $$I\left(\%\right)=100-[(A_{s}-A_{b})\times \frac{100}{A_{c}}]$$

I (%) represents the inhibition percentage. The spectrophotometer underwent calibration using ethanol, with (A_s_) denoting the absorbance values corresponding to diverse solution concentrations. As a blank reference (A_b_), a solution comprising 2 mL of ethanol and 2 mL of Pro solutions at different concentrations was utilized, and the blank’s absorbance was measured for each concentration. The control solution was prepared using 2 mL of ethanol and 2 mL of DPPH solution. The IC50 value, expressed in µg/mL, was calculated from the graphical depiction of I% against concentration, signifying the concentration of the sample required to neutralize 50% of the free radicals in the DPPH solution.

## 3. Results and Discussion

### 3.1. FTIR Spectroscopy of SA and OSA

**Figure 4:**
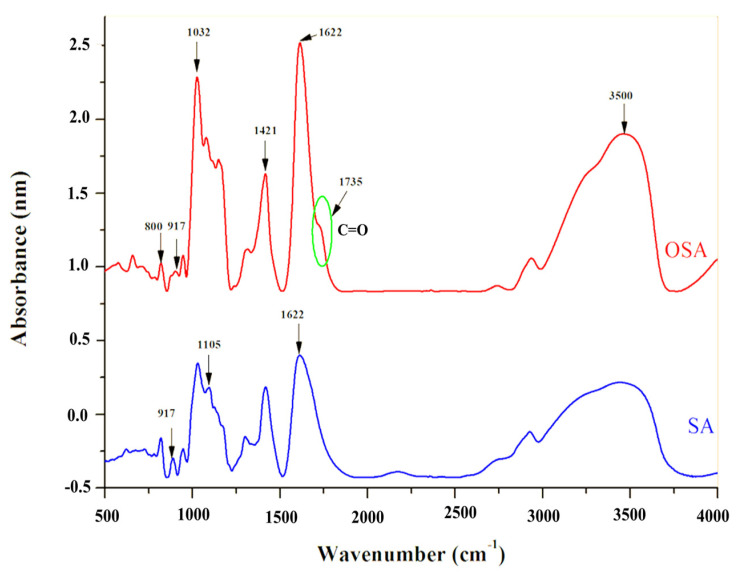
Fourier-transform infrared (FTIR) spectra of SA and OSA (oxidation time: 24 h) the green circle clarified the C=O bound.

The FTIR analysis was carried out to detect the appearance of new chemical bonds or the modification of existing ones, which could demonstrate the oxidation of SA and the presence of aldehyde groups in the OSA. Figure 4 shows the infrared spectra of SA and OSA after 24 h of oxidation:

The infrared spectra of the alginate present the typical bands of its polysaccharide structure. The broad absorption peak shown at 3465 cm^−1^ is assigned to the stretching vibration of hydroxyl groups (–OH) [22,23,24,25]. The absorption bands at 2943, 1031, and 1105 cm^−1^ are typical peaks of all polysaccharide derivatives. These peaks can be attributed to the C–H stretching vibration elongation of the C–O–C groups and C–C stretching groups, respectively [49,50,51]. The absorption peaks at 1622 and 1421 cm^−1^ are related to the asymmetric and symmetric carboxylate salt groups of alginate [22,23,24,25,26,27,28]; the figure also shows a peak at 1312 cm^−1^ due to single bond stretching vibration in C–O groups [52]. Compared to the SA infrared spectra, a new characteristic band is shown at 1735 cm^−1^ in the FTIR spectra of OSA; it is assigned to the carbonyl signal peaks of the aldehyde group –C=O, which confirmed the occurrence of the oxidation reaction [24,29,49,50,52,53,54,55,56,57]. This peak was weak in this study and, in some studies, is not detected due to the formation of intermolecular hemiacetals structures between the free aldehyde groups and neighboring hydroxyl groups on the adjacent uronic acid [24,49,58,59].

Moreover, the stretching vibration frequency of the absorption peak of –C–O bonds shifted from 1314 cm^−1^ in the SA spectrum to 1352 cm^−1^ in the spectrum of OSA [52]. These results indicated the cleavage of –C2–C3– bonds between vicinal glycols on alginate uronic acid residue by the oxidation reaction [27,49,52]. In addition, the similarity of infrared spectroscopy results of the OSA and native SA demonstrated that the periodate cleaved only the –C2–C3– bond by the oxidation reaction [47]. Absorption bands from about 800 cm^−1^ can be attributed to hemiacetals formed between the aldehyde group and vicinal hydroxyl groups in an acidic medium, but they could also occur in a neutral medium.

### 3.2. NMR Spectroscopy of SA and OSA

**Figure 5:**
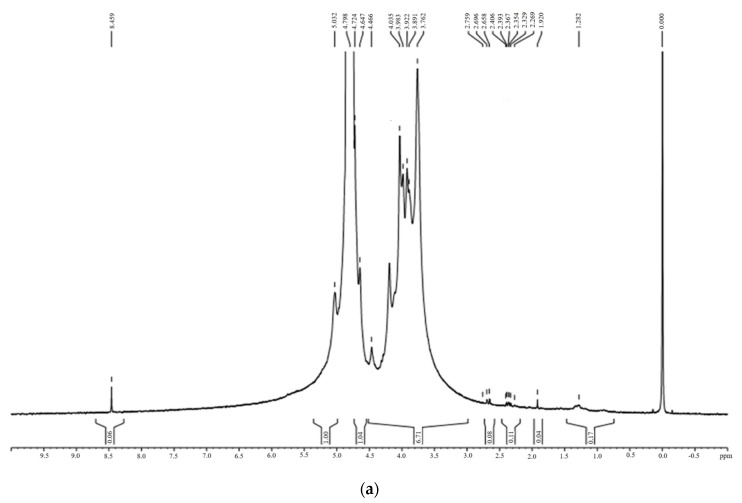

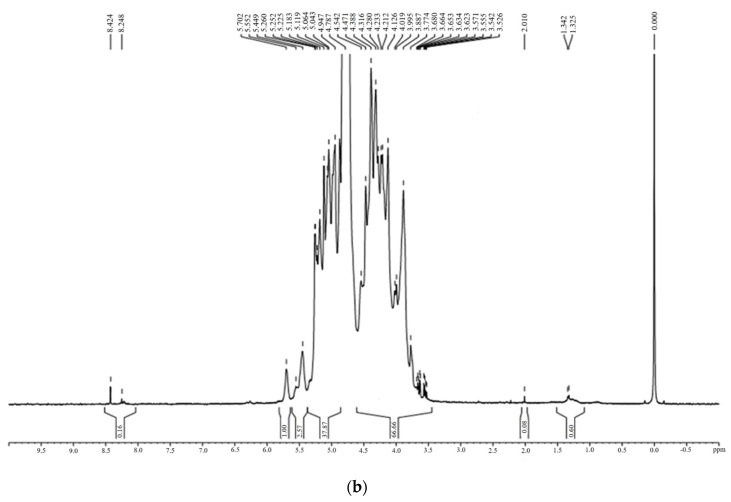
Proton nuclear magnetic resonance (^1^H NMR) spectra for SA dissolved in water (**a**) and OSA dissolved in D_2_O (**b**).

The oxidation of sodium alginate was confirmed by the comparison of ^1^H NMR spectra of SA and OSA using D_2_O as a solvent (Figure 5):

The ^1^H NMR spectra of SA exhibited ranging peaks from 3.762 to 5.0 ppm, corresponding to the anomeric proton of Glucuronic (G) and Mannuronic (M) units [60]. The peaks at 5.0 and 3.7 ppm were assigned to the G-1 and M-5 protons, respectively. Meanwhile, the peaks between 4.66 and 4.798 ppm corresponded to the G-5 and M-1 protons [60,61,62]. Compared to the OSA ^1^H NMR spectra, a new peak at 8.24 ppm appeared, which was attributed to the proton of the aldehyde groups –CHO [54,58,60]. Two signals at 5.449 and 5.702 ppm were assigned to the protons in hemiacetals structures [28,52,55,56,63]. These structures resulted from the aldol condensation reaction between the aldehyde and contiguous hydroxyl groups in the D_2_O solvent [60,64,65].

Moreover, the intensity of the peaks at 3.7 and 5.0 ppm that corresponded to the signals of protons of M-5 and G-1 decreased, and the signal of the G-5 proton was modified. The above results confirmed the oxidation of sodium alginate using NaIO_4_ and the change in surroundings caused by the cleavage of the vicinal –C2–C3– bonds [66]. In some cases, the peak of aldehyde groups did not appear due to the equilibrium of the aldehydes in water with their hydrated forms, and they react with the unreacted hydroxyl groups of the SA chain [62].

### 3.3. Oxidation Reaction Kinetic of SA

**Figure 6:**
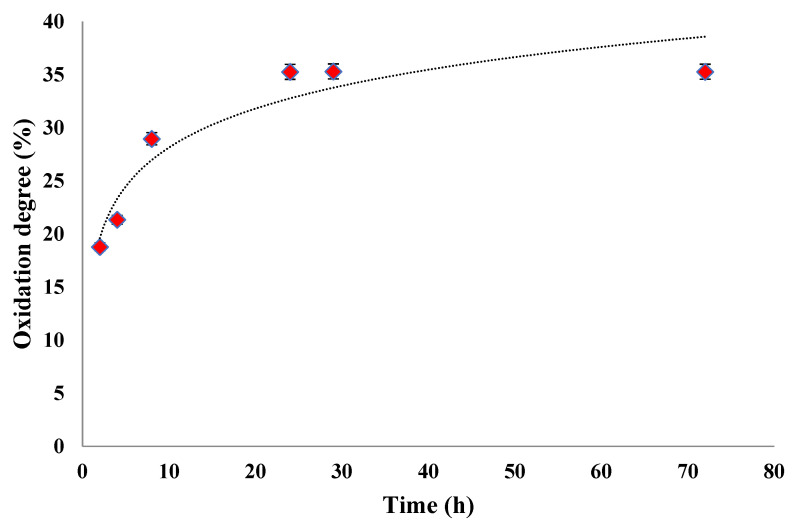
Variation in time of the oxidation degree of SA.

The kinetics of the SA oxidation in the presence of NaIO_4_ were studied by determining the consumption of sodium periodate during the reaction. Figure 6 shows the obtained results.

SA was oxidized in the dark in an aqueous solution using sodium periodate as an oxidizing agent at room temperature for 24 h. Different reaction times were used to determine the maximum oxidation degree value and optimize the oxidation reaction parameters (Figure 6). The sodium periodate cleaved the vicinal dihydroxyl groups in the SA chains at C2–C3. The aldehyde groups were generated, and the degradation of the polymer occurred [27,28].

The result shows that the oxidation degree gradually increased with time until 24 h had passed, reaching a value of 35.26%. After 24 h, the oxidation degree value was maintained at an almost constant until 72 h, meaning that not all the vicinal hydroxyl groups were oxidized, and the SA was only partially oxidized. Similar results, in which the oxidation degree increases over time up to 24 h, can be found in the literature [54].

The maximum degree of oxidation varied between 35% and 50% due to the formation of stable inter-residue hemiacetals between an aldehyde group and a hydroxyl group in the monomeric units, resulting in the protection of the hydroxyl group from further oxidation [22,67,68,69].

### 3.4. Temperature Influence on the Oxidation Degree

**Figure 7:**
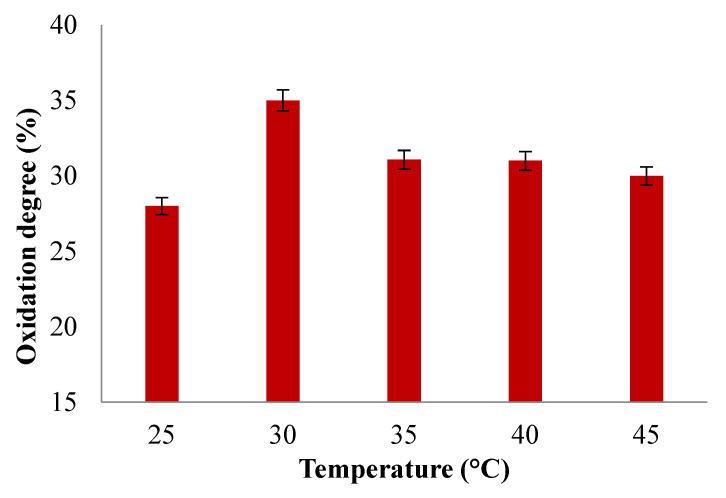
Variation of SA oxidation degree as a function of temperature (t = 24 h).

The effect of temperature on the degree of oxidation of SA was studied by keeping the duration of the reaction constant (t = 24 h). Figure 7 shows the results obtained.

In order to study the effect of temperature on the oxidation reaction, different oxidation degrees were determined at various reaction temperatures varying from 25 °C to 45 °C (Figure 7). The results show that the oxidation degree was higher when the temperature increased to 30 °C, reaching an oxidation degree of 35%. Increasing the temperature up to 45 °C led to a decrease in oxidation degree to 30%, diminishing the oxidation degree, which is in agreement with the decomposition of periodate, which affects the aldehyde groups yield more or less if the oxidation reaction occurs at temperatures over 45 °C, as already reported by Chemin et al. and Li et al. [70,71]. At higher temperatures, the periodate ion (IO^4−^) can undergo a series of reactions that result in the formation of iodate (IO^3−^) and oxygen (O_2_) gas. This process can lead to incomplete or inefficient oxidation and reduces the effectiveness of periodate as an oxidant in the reaction, which can induce lower yields or undesirable byproducts.

### 3.5. FTIR Spectroscopy of the Hydrogels

**Figure 8:**
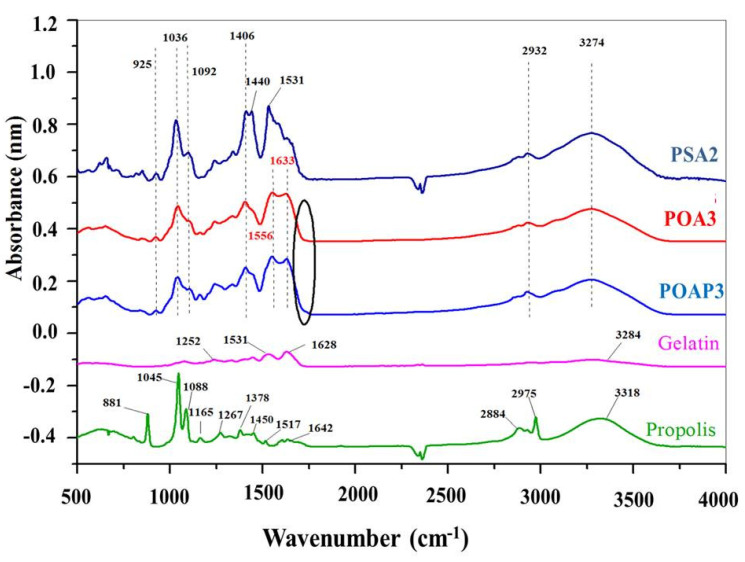
FTIR analysis of hydrogels (PSA2 SA/G 1:1, POA3 OSA/G 1:1, POAP3 OSA/G/Pro hydrogels), Gel, and Pro.

Figure 8 illustrates the FTIR spectra of Gel, Pro, PSA2, POA3, and POAP3 hydrogels at a pH of 5.5 (with and without Pro) to establish the new absorption bands.

The FTIR spectrum presented in Figure 8 reveals distinctive absorption bands corresponding to the protein structure of the Gel. Notably, the peaks from 1628, 1531, and 1250 cm^−1^ are assigned to the –N–H stretching vibration peaks for amide I, amide II, and amide III [72,73]. On the other hand, the bands around 1036 and 1092 cm^−1^ refer to the -C–O and –CO–C vibration of groups in mannuronic and guluronic units, respectively, and the band at 925 cm^−1^ is assigned to the –C–O vibration of groups in the A-configuration of the guluronic units. These bounds are attributed to the saccharide structure of SA [74]. The bands at 1421 cm^−1^ are attributed to –COO– (in the SA and OSA, Figure 4), shifted to a lower wavenumber in the hydrogel samples to 1406 cm^−1^, confirming the ionic cross-linking of SA with Mg^2+^ [75]. Moreover, the absorption band at 3274 cm^−1^ could be attributed to the stretching vibration of the –O–H group (from the SA or OSA) bonded to the –N–H group (from the Gel). The peak of this band in the SA and OSA was at the wavenumber of 3500 cm^−1^ and 3284 cm^−1^ in the Gel spectrum, respectively. The maximum intensity of this bond exhibited a reduction in polymers compared to the spectra of the individual polymer, proving the interaction of this group in the cross-linking reaction [75,76].

FTIR spectra of the POA3 and POAP3 hydrogels verified the interactions between the carbonyl group from the OSA and the amino groups from the Gel to produce the Schiff base cross-linking. The presence of –N=C– bonds confirms the realization of the cross-linking reaction. In Figure 8, the POA3 and POAP3 spectra present two intense bonds at 1556 and 1633 cm^−1^ due to the –N=C– stretching, which suggests the formation of the Schiff base bonds [77,78,79,80]. The band’s broadening at 1633 cm^−1^ attributed to Schiff’s base is likely a result of overlap with the band at 1628 cm^−1^ corresponding to amide I of uncrosslinked Gel [79]. Furthermore, the absorption peak corresponding to the aldehyde group in OSA at 1735 cm^−1^ vanished, and a distinct peak emerged at 1633 cm^−1^, indicative of a –C=N– double bond leading to the formation of a hydrogel network [81].

The FTIR spectrum of Pro showed the presence of phenolic compounds or their esters (–O–H at 3318 cm^−1^, –C–O– at 2884 cm^−1^, and –C–H aromatic at 2975 cm^−1^) [80]. The peaks observed in the range of 1642 cm^−1^ are attributed to the –C=O and –C=C– stretching vibrations of flavonoids, as well as the –N–H asymmetric stretching of amino acids. Furthermore, a strong agreement is noted between the signals in the analyzed sample and the literature [82] for phenols and flavonoids. This correspondence serves as a robust indicator of the presence of both types of compounds in the extract. The characteristic signals for these compounds include stretching and bending vibrations at 1450 cm^−1^, vibrations and bending at 1378 cm^−1^, and vibrations and bending at 1088 cm^−1^. The peak at 1267 cm^−1^ is attributed to the hydrocarbons’ asymmetrical –O–H and –C–CO bending. The peaks at 1043 cm^−1^ and 880 cm^−1^ are also associated with primary and secondary alcohols. Specifically, ethanol exhibits a symmetrical stretching at 881–880 cm^−1^, aligning with the fact that the extracts are dissolved in ethanol [83]. Moreover, the minor peak at 1517 cm^−1^ might be attributed to the flavonoids or aromatic ring, and the bound at 1165 cm^−1^ corresponds to the lipid or the tertiary alcohol groups [84].

### 3.6. Thermogravimetric Analysis (TGA)

**Figure 9:**
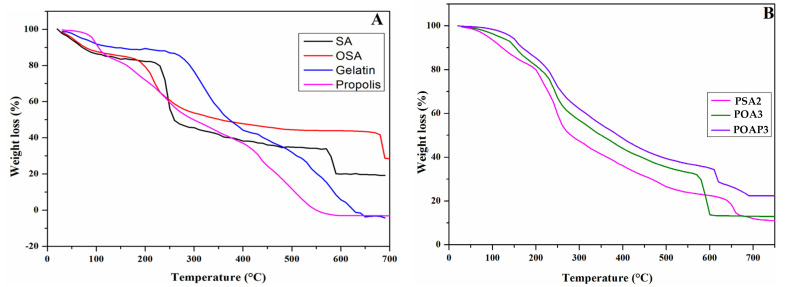
Thermogravimetric analysis curves of (**A**) SA, OSA, Gel, and Pro; (**B**) hydrogels.

Figure 9 presents the thermogravimetric analysis of pure materials (SA, Gelatin, propolis) and OSA, as well as PSA2, POA3, and POAP3 hydrogels. The main data are summarized in Table 2.

TGA is a robust method for evaluating the thermal stability of composite hydrogels. It provides a foundation for indirectly investigating the interactions among the components of these hydrogels. This is achieved by comparing key parameters, such as the final mass loss, the rate of weight loss at the final stage, and the Tmax, which refers to the temperature at which the maximum rate of weight loss occurs during the thermal decomposition across different samples. Figure 9A shows that the thermogravimetric curve of Gel displays three stages of thermal degradation. The first stage of weight loss is perceived across the temperature range of 38–115 °C, accompanied by a weight loss of 7.86%, possibly due to the release of free-binding water and volatile component loss. In the second stage, 45.68% weight loss is observed in the temperature range of 230–420 °C, related to the decomposition of molecules with low molecular weights in the Gel [85].

Between temperatures of 470 to 660 °C, the third phase of weight reduction became evident, with a weight loss of 36.32%, referring to the complete thermal decomposition of Gel chains [80]. On the other side, from the TGA curve of Pro, it can be appreciated that the Pro thermal stability sample was assessed by three main degeneration stages, where the significant decrease observed at the temperature of 440 °C indicated a substantial reduction in the weight of the samples. The first degradation stage occurred in the temperature range between 40 and 130 °C, with mass losses of approximately 15.38%. This indicated the breaking of hydrogen bonds, subsequently releasing water molecules and other volatile compounds with low molecular weight [86,87,88].

Besides SA and OSA, the TGA curve also illustrates three phases. From Table 2, the first phase saw 13.45% and 9.33% weight loss for SA and OSA at temperatures below 100 °C due to the evaporation of free and bound water in the samples [89]. At 170–300 °C, significant weight reductions of about 37.15% and 30.54% were observed for SA and OSA, respectively, which might be attributed to the dehydration of hydroxyl groups along the alginate backbone and the thermal decomposition of mannuronic acid (M) and guluronic acid (G) residues, known as hexuronic acid segments [52]. In the temperature range between 560 and 700 °C, due to increased carbonization decomposition and decarboxylation of the residues [90] as compared to the Gel and SA, OSA exhibited a higher residual fraction (28.71%) and a lower degradation rate. Moreover, the temperatures at which the maximum rate of weight loss occurred during the thermal decomposition were 240 °C and 219 °C for SA and OSA, respectively, and all these results confirmed the higher thermal stability. We determined from the results that oxidized functional groups, such as carbonyl groups, may contribute to increased thermal stability by introducing stronger chemical bonds and modifying the overall molecular structure. These modifications can make the polysaccharide more resistant to thermal decomposition. During the second stage, 33.08% weight loss was observed at a temperature range of 140–300 °C, which might have contributed to the degradation of phenolics, carbohydrates, and amino acids [86,88,91]. At higher temperatures, the elimination of other organic compounds occurred. This process is minorly related to the combined decomposition of amino acids and fiber, continuing until the final residues are obtained [85,86]. On the other hand, it can be seen from Figure 9B that the hydrogels TGA curve of all hydrogels showed three stages, with the first one at approximate temperature ranges of 53.18–164 °C, 130–190 °C, and 130–181 °C with weight losses of 14.01%, 11.08%, and 8.21% for PSA2, POA3, and POAP3, respectively. These temperature ranges correspond to the removal of adsorbed and bound water and the loss of volatile compounds. The second stage was in the temperature ranges of 180–295 °C, 200–285 °C, and 200–280 °C with weight losses of 35.24%, 20.35%, and 19.99% for PSA2, POA3, and POAP3, respectively, which might describe the degradation of peptide bonds within protein chains and the hydroxylic gropes within the hydrogels. The third stage for PSA2 hydrogel occurred in the 611–680 °C range with a weight loss of 10.15%, due to the decomposition of the Gel residual chain. However, for POA3 and POAP3 samples, the decomposition occurred between 557 and 610 °C and 600 and 690 °C for POA3 and POAP3, respectively, with weight losses of 19.1% and 13%, which contributed to the decomposition of thermally stable structures such as imine bonds. Table 2 shows that the PSA2 hydrogel presented a lower weight loss rate with a lower final fraction residual of 10.55% compared to the POA3 (13%) or POAP3 (22.39%) Schiff base-based hydrogels. Moreover, the Tmax reduced with the introduction of the aldehyde groups into the hydrogel matrix, which means that the PSA2 exhibited a higher Tmax (250 °C) compared to the POA3 (240 °C) and POAP3 (237 °C) hydrogels. Therefore, the Schiff base hydrogels POA3 and POAP3 demonstrated greater stability against thermal degradation than the SA/Gel-based hydrogel PSA2, suggesting that the cross-linking of Gel/OSA with an imine bond significantly improved the thermal stability [90]. Moreover, the addition of Pro within the OSA/Gel matrix hydrogel makes it more thermally stable, as confirmed on the sample POAP3.

### 3.7. Scanning Electron Microscopy (SEM) Analysis

SEM analysis was conducted to visualize the film’s surface and cross-section morphology. To demonstrate how the incorporation of OSA in the film matrix and the addition of Pro affected the morphology of the hydrogel films, we selected PSA2, POA3, and POAP3 samples for this aim. The molar ratios of –CH=O/–NH_2_ used to obtain the films were 1:1 for both samples, adding 0.5 mL of Pro to the POAP3 sample.

**Figure 10:**
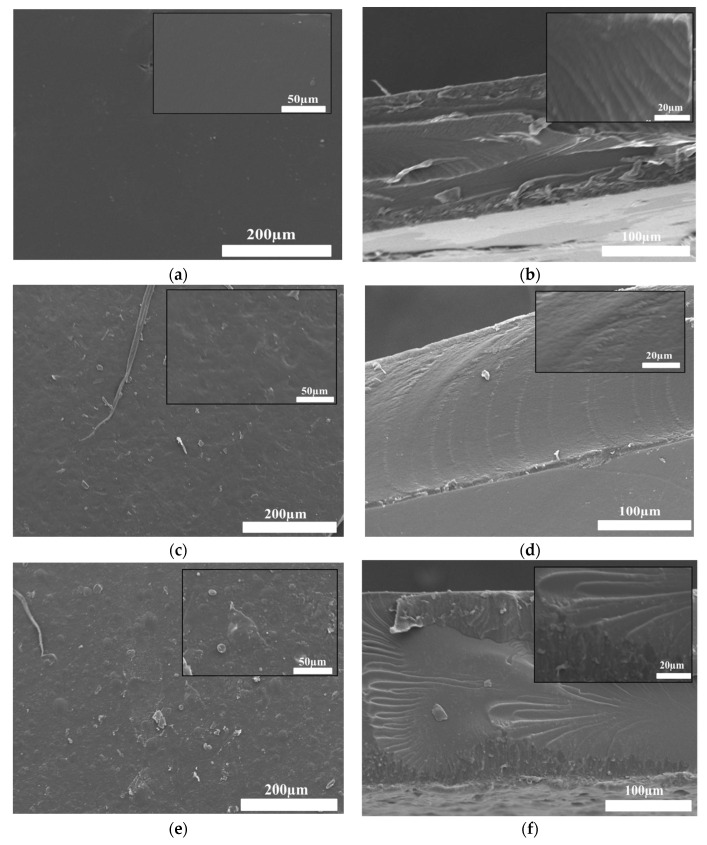
Scanning electron microscopy photographs of the samples. (**a**) PSA2 surface; (**b**) PSA2 cross-section; (**c**) POA3 surface; (**d**) POA3 cross-section; (**e**) POAP3 cross-section; and (**f**) POAP3 cross-section.

Figure 10 displays SEM surface images as well as the cross-section for SA/Gel, OSA/Gel, and OSA/Gel/Pro hydrogels to evidence the effect of cross-linking by Schiff base links, as well as the introduction of the Pro in the hydrogel matrix. It can be seen that the roughness of the hydrogel surface increased with the addition of the OSA and the Pro in the hydrogel matrix. The SA/Gel PSA2 hydrogel showed a compact, smooth, continuous, flat surface morphology. However, the OSA/Gel base hydrogel surface exhibited a rough, dense, and slightly porous morphology. Incorporating Pro into the hydrogel matrix increased the roughness of the hydrogels.

The cross-section morphology of the native PSA2 was compact and lacked any distinctive pore structure. On the other hand, the OSA/Gel and OSA/Gel/Pro composite hydrogels indicated less compact morphology. As illustrated in Figure 10f, the incorporation of Pro into the OSA/Gel matrix was evident in a more densely packed structure, as was also shown in another research study [80].

### 3.8. Amino Groups Conversion Index Determination

The number of moles of free amino groups from the Gel was determined using the ninhydrine test. This information was essential in order to prepare the hydrogel films. We used different molar ratios between the free amino groups from Gel and aldehyde groups from OSA.

Based on the glutamic acid calibration curve and the ninhydrin test, the free amino groups presented in the native Gel (found at 1.69 × 10^−3^ mol/g) and in the obtained hydrogels were determined. Hence, by the difference between the number of initial free amino groups in Gel and those determined in the films, the conversion index of amino groups obtained in Schiff’s bases in hydrogel films was established based on Equation (2) from methods. Table 3 shows the values of the conversion index for the hydrogels in correlation with Pro’s encapsulation efficiency.

**Figure 11:**
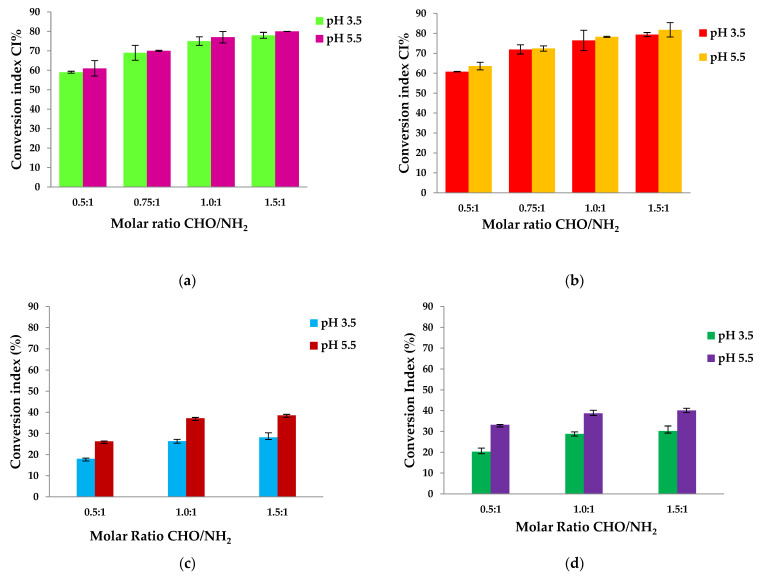
The variation in the amino group’s conversion index values as a function of the molar CHO/NH_2_ ratios and pH (t reaction = 6 h, T reaction = 37 °C). (**a**) Samples with OSA and without propolis obtained at pH 3.5 and 5.5; (**b**) samples with OSA and with propolis obtained at pH 3.5 and 5.5; (**c**) samples with SA and without propolis obtained at pH 3.5 and 5.5; and (**d**) samples with SA and without propolis obtained at pH 3.5 and5.5.

The average percentage of free amino groups after cross-linking reactions at different pH (3.5 and 5.5) and different molar ratios CHO/NH_2_ was determined by using the ninhydrine test. The results are shown in Figure 11.

The hydrogels were prepared using different molar ratios between the amino groups from Gel and the aldehyde groups from OSA. The number of moles of amino groups from Gel was maintained constant for all the samples, namely 3.38 × 10^−3^ moles, and the number of moles of carbonyl groups from OSA was varied by variation in OSA amount. The pH levels of the media in which the biopolymers were dissolved before obtaining the hydrogel were 3.5 and 5.5. The magnesium ions were added to the OSA solution before adding the Gel to avoid the ionic complexation between the Gel and OSA. Figure 11 shows that the CI values increased when the –CHO/–NH_2_ molar ratio increased from 0.5:1 to 1.5:1. The pH medium’s value did not significantly influence the CI values obtained. It increased from 59% to 78% for pH = 3.5 and from 61% to 80% for pH = 5.5. The difference between the values obtained at pHs = 3.5 and 5.5 were small and statistically insignificant. This means that the degree of cross-linking increased as the amount of OSA increased because the reaction of aldehyde groups facilitates hydrogel formation [63,83]. The IC values of hydrogels with immobilized active ingredients increased from 60% to 79% at pH = 3.5 and from 63% to 83% at pH = 5.5; these values are close to those obtained without propolis. The difference observed between samples with propolis and those without was minimal and has no statistical significance. This suggests that, although there is a slight variation, propolis components do not interact significantly with gelatin amino groups. Although minor interactions, such as hydrogen bonds, may occur, these are likely to be disrupted at the reaction temperature of 100 °C, where the ninhydrin reaction takes place, meaning that the presence of propolis has no significant effect on the interaction with gelatin under these conditions.

Because Pro contains various compounds, they may interact with amino acids and protein groups from Gel to form covalent bonds, hydrogen bonds, and electrostatic interactions, slightly increasing the CI values.

On the other hand, the aldehyde groups within OSA might form hemiacetals with the hydroxyl groups. Thus, not all the resulting aldehyde groups are available to react with the amino groups from the Gel to form Schiff bases (or imine bonds), and this could describe the incomplete reactivity of amino groups even in the case of excess aldehyde groups theoretically presented in the OSA. Moreover, it must be noted that some intermolecular interactions exist, such as hydrogen bonds, which could block the free amino groups [44].

Another parameter affecting the cross-linking reaction is the pH of an acidic medium. Figure 5 shows that the conversion index at pH = 3.5 is slightly lower than at pH 5.5. In both acidic media, protonated amine groups in the gel facilitate the crosslinking reaction with aldehyde groups [92]. The isoelectric point of Gel type A is found at a pH value between 7 and 8.5. At this pH, the charge of the protein is neutral, and its functional groups do not react. The literature mentions rapid gelation before the isoelectric point until pH = 7, where the amino groups are protonated [93]. A negative charge could be generated on the Gel backbone above the isoelectric point because the proteins have an amphoteric character [94]; in this pH range, the substituting –COOH groups on the main chain of the protein are found in dissociated form as carboxylate ions. The pKa of the carboxyl groups is at pH 3.5. At this pH, electrostatic interactions between the carboxyl groups of the OSA and the protonated amine groups of the gel did not occur. The CI values are slightly higher at pH = 5.5 because, at this pH value, the free amino groups within Gel may react not only with the aldehyde groups but also with the carboxylic groups that did not participate in the ionic cross-linking with Mg^2+^ to form polyelectrolyte complexes. Since steric hindrances can occur between macromolecules, it is not possible to obtain a 100% conversion index of amino groups to Schiff bases, even if a –CHO/–NH_2_ molar ratio of 1.5:1 (e.g., an excess of –CHO groups) was used.

To elucidate the specific Schiff base cross-linking within the film and explore the potential formation of electrostatic interactions between amino groups from gelatin and –COOH groups from OSA at two different pH levels, twelve films (six without Pro and six with Pro) were studied. The free amino groups were quantified by dosing them with ninhydrin after drying the hydrogel films. The results are illustrated in Table 4 and Figure 11c,d. It is important to note that the films were prepared with sodium alginate to facilitate this test.

It should be noted that the CI (%) values are annotated as follows:

CI chemical crosslinking + physical interaction (%) represents the overall involvement of amino groups in both chemical bonds and physical interactions.

CI physical interactions (%) indicates the percentage of amino groups exclusively involved in physical interactions.

CI chemical crosslinking + physical interaction − CI Physical interaction = CI chemical cross-linking (Schiff base) (%), exclusively involving the Schiff base interaction between the amino and the CHO groups.

It was found that in non-chemically cross-linked samples, some of the amino groups from Gel did not participate in the reaction with ninhydrin, obviously because –NH_2_ groups were involved in stable interactions with carboxylic groups of SA. It is assumed that the same effect occurs when cross-linked samples are obtained, i.e., some of the amino groups of Gel react with the OSA carbonyl groups, and others interact with the carboxylic group of OSA. These interactions could explain the high CI values corresponding to these samples, which do not correctly express the CI of amino groups into Schiff bases.

Samples obtained at pH = 3.5, such as the POB1 sample, had a Schiff base % formation of 41.35% (as shown in Table 4). The maximum theoretical value for the Schiff bases reaction, based on the molar ratio of the functional groups in the PB1 sample, is 50% (0.5 moles of –CH=O for 1 mol of -NH_2_). For the POB2 sample obtained at a molar ratio of 1:1, the Schiff base formation was 48.67%. POB3 was obtained at a molar ratio of 1.5:1, and the Schiff base formation was 50.39%. At pH = 5.5, the Schiff base formation for the POA1 (CHO/NH_2_ = 0.5/1) sample was 34.79%. Similarly, for the POA2 (CHO/NH_2_ = 1/1) sample, there was a significant difference of 40.82%. For the POA3 samples, obtained at a molar ratio of 1.5 moles of –CHO groups for 1 mol of –NH_2_ groups, the Schiff base formation was 42.09%. The observed difference in physical cross-linking between amino groups from Gel and carboxylic groups from SA in samples prepared at pH 3.5 versus those at pH 5.5 is likely influenced by several factors. It was also observed that not all the amino groups reacted due to the steric hindrance. It was observed that when the molar ratios increased, the amount of oxidized sodium alginate increased, and electrostatic interactions occurred.

The difference observed in the physical cross-linking between Gel amino groups and SA carboxyl groups in samples prepared at pH 3.5 compared with those prepared at pH 5.5 is probably influenced by several factors. At pH values of 3.5 or 5.5, the NH_2_ groups of gelatin are protonated, as these pH values are below the isoelectric point of gelatin. At pH values above 3.5 (the pka for COOH groups), carboxyl groups can be deprotonated, forming carboxylate ions that can be found in oxidized alginate in a large number as the pH value of the cross-linking reaction medium increases. These carboxylate groups can react with amino groups instantly, leading to the formation of polyelectrolyte complexes and preventing the formation of Schiff bases, which occurs after a few hours. Generally, the carboxylate groups of anionic polysaccharides are fully deprotonated under basic pH conditions, increasing their reactivity in such complex-forming processes.

The results confirm that the Schiff base cross-linking for the samples obtained at pH 5.5 was slightly lower than those at pH 3.5, confirming that although pH influences cross-linking, the variation in pH does not significantly affect the Schiff base formation between the gelatin and OSA groups.

### 3.9. The Molar Ratio –CHO/–NH_2_ Influences on the Swelling Degree

**Figure 12:**
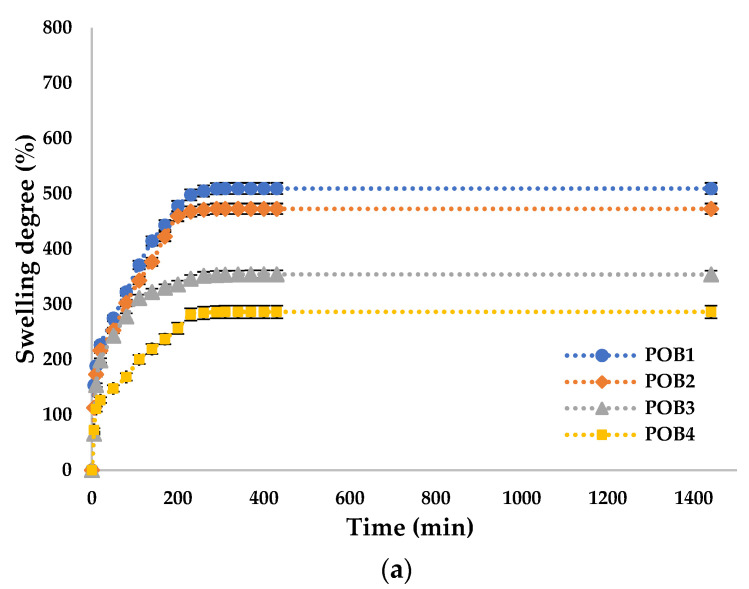

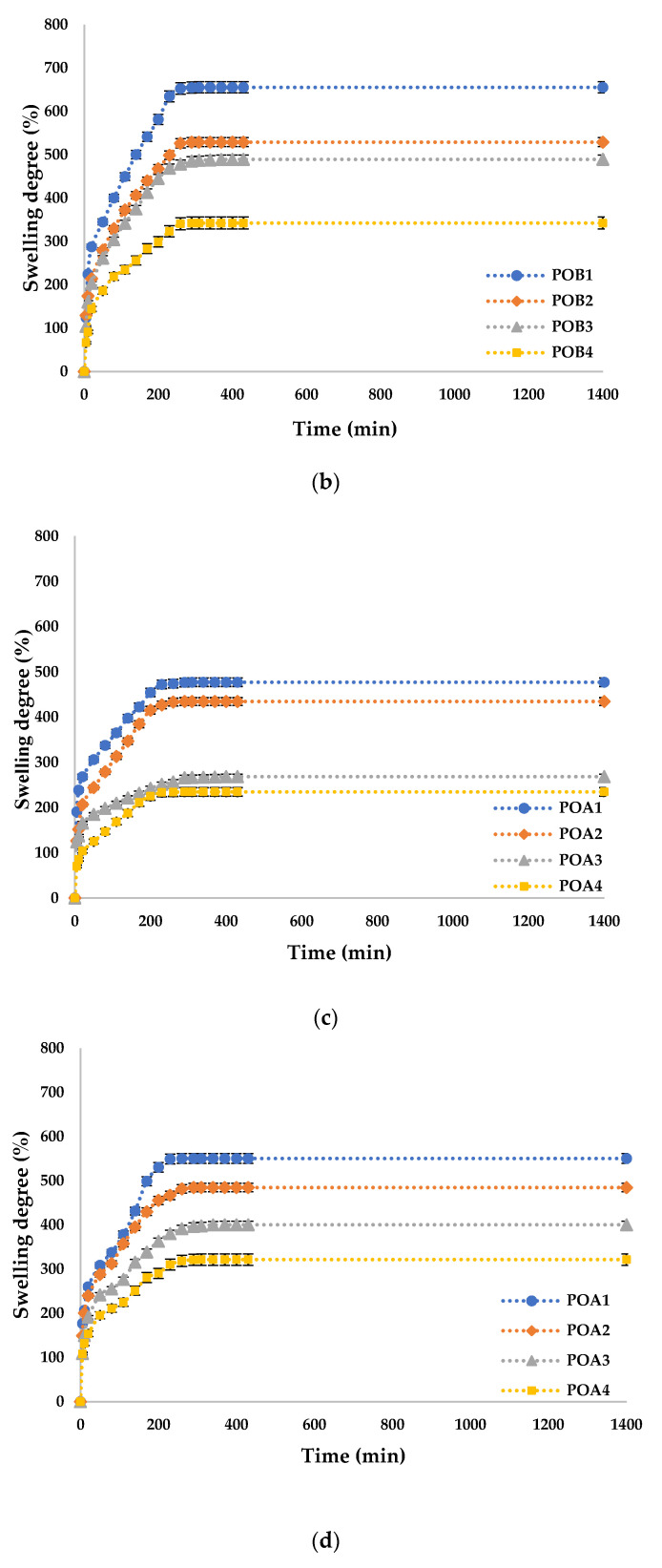
The swelling degree kinetics at different –CHO/–NH_2_ molar ratios of (**a**) hydrogels prepared at pH = 3.5 immersed in acetate buffer solution (ABS) at pH = 5.5; (**b**) hydrogels prepared at pH = 3.5 immersed in phosphate buffer solution (PBS) at pH = 7.4; (**c**) hydrogels prepared at pH = 5.5 immersed in acetate buffer solution at pH = 5.5; and (**d**) hydrogels prepared at pH = 5.5 immersed in phosphate buffer solution pH = 7.4.

**Figure 13:**
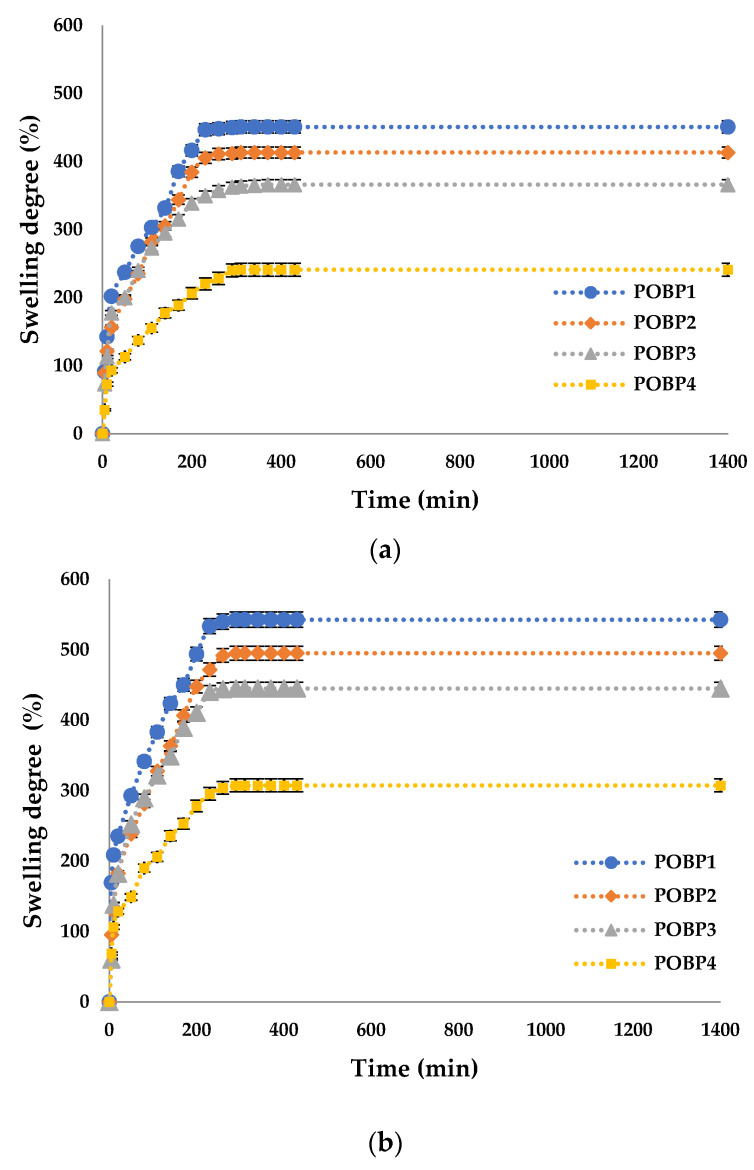

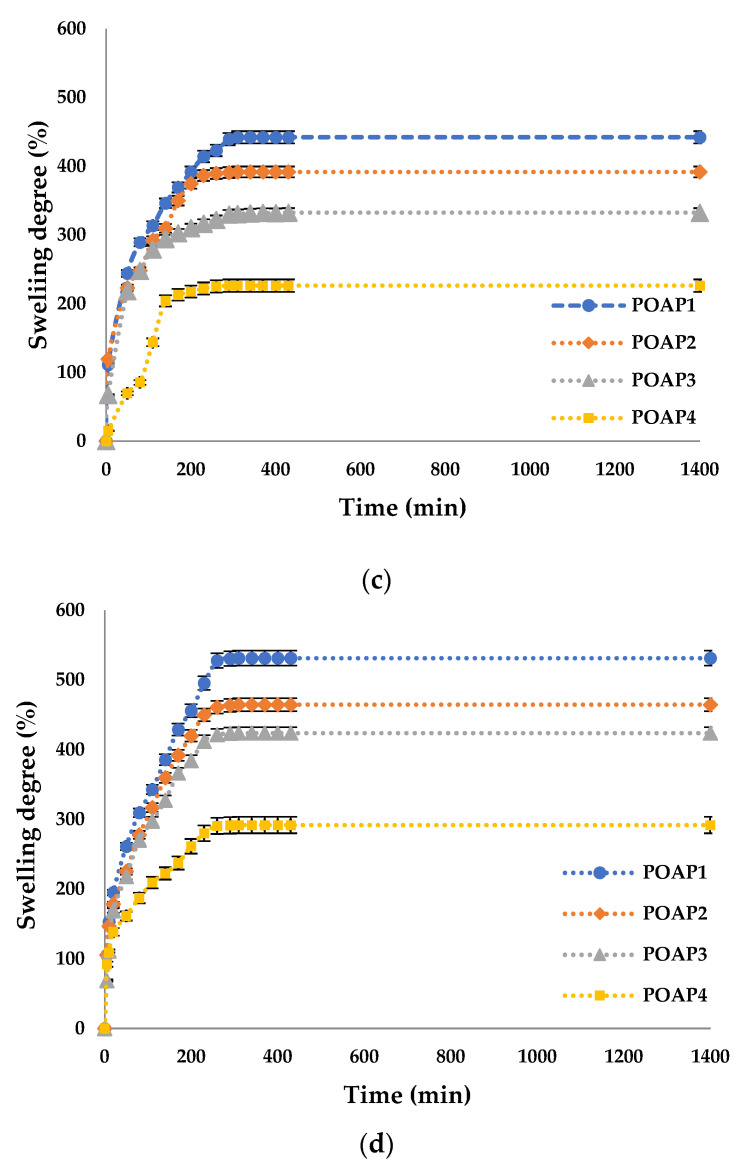
The swelling degree kinetics at different –CHO/–NH_2_ molar ratios of (**a**) hydrogels prepared at pH = 3.5 with Pro immersed in acetate buffer solution (ABS) at pH = 5.5; (**b**) hydrogels prepared at pH = 3.5 with Pro immersed in phosphate buffer solution (PBS) at pH = 7.4; (**c**) hydrogels prepared at pH = 5.5 with Pro immersed in acetate buffer solution at pH = 5.5; and (**d**) hydrogels prepared at pH = 5.5 with Pro immersed in phosphate buffer solution pH = 7.4.

The swelling degree (Q %) was determined to evaluate the capacity of the OSA/G hydrogels to absorb and retain the aqueous solution at different pH mediums. The Q% is an essential property of hydrogels with biomedical applications because the diffusion of the encapsulated bioactive principle from the polymer matrix depends on it. The swelling degree of the hydrogels with/without propolis, with different –CHO/–NH_2_ molar ratio values, was measured until equilibrium in two different pH mediums (pH = 5.5 and pH = 7.4) at 37°C. The results are shown in Figure 12 and Figure 13.

Figure 12 shows that Q% values were higher at pH = 7.4 than at pH = 5.5 because the hydrogels contained OSA in their structure. Therefore, the basic pH induced the formation of carboxylate anions from the acid groups that did not participate in the cross-linking with Mg^2+^ ions or in interactions with amino groups, which led to the electrostatic repulsions between the polysaccharide chains and had an effect on the relaxation of the network, facilitating the diffusion of higher amounts of water.

The isoelectric point of Gel type A is found at around pH = 7.4. At this pH, the interactions of the functional groups within the protein are weak, and the hydrogen bonds do not form. At pH = 5.5, several hydrogen bonds could be formed in the hydrogel films, leading to lower adsorption of the swelling medium. Consequently, these intermolecular attraction forces predominantly have polymer–polymer interactions rather than medium–polymer interactions, resulting in a smaller amount of medium absorbed by these hydrogels [95].

The variation in the degree of swelling of the material is inversely proportional to the degree of crosslinking, meaning that as the degree of crosslinking increases, the degree of swelling decreases. This relationship is influenced by the molar ratio of amino groups to carbonyl groups used in cross-linking. Carboxyl groups in oxidized sodium alginate significantly influence the swelling behavior of the hydrogels prepared. They introduce additional hydrophilic sites, increasing the hydrogel’s capacity to absorb water. These results were confirmed in the work of Baron et al. [96] and Tincu (Iurciuc) et al. [46], which demonstrated similar findings. These results align well with the conversion index, confirming the expected behavior of the material under varying cross-linking conditions.

Figure 13 shows that adding Pro leads to a slightly decreased swelling degree compared to the gels that do not contain the active principle. The active principle is located within the pores of the polymer matrix, reducing the aqueous solution’s absorption. Additionally, propolis is poorly soluble in water and can make the film hydrophobic, leading to lower aqueous solution absorption and, consequently, a lower degree of swelling.

### 3.10. Encapsulation Efficiency

As previously mentioned, propolis’s encapsulation and release efficiency have been indirectly assessed through the encapsulation and release of the p-coumaric acid it contains.

**Figure 14:**
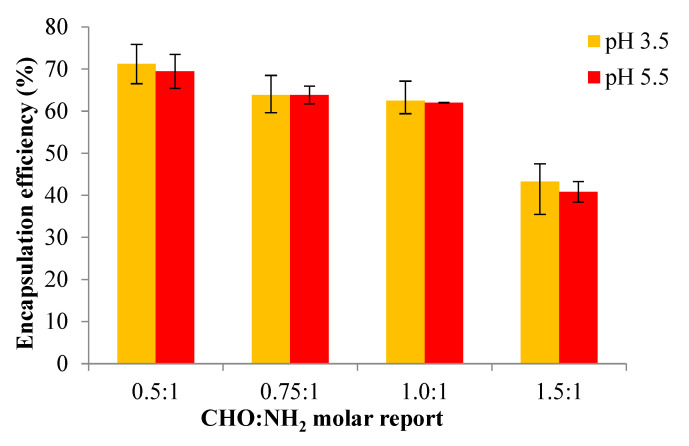
Pro encapsulation efficiency in OSA/Gel-based hydrogels obtained at different pH mediums and different –CHO/–NH_2_ molar ratios.

Figure 14 presents the results obtained for the encapsulation efficiency of Pro for the OSA/Gel-based hydrogels prepared in two different pH mediums. Due to the biocompatibility of the biopolymers used in the OSA/Gel-based hydrogel preparation, this study further aims to confirm their feasibility for the encapsulation and release of active compounds. The selection of Pro as a drug model involved assessing its encapsulation efficiency within OSA/G-based hydrogels, which was determined by evaluating the presence of p-coumaric acid as a crucial component of Pro [The volume of Pro solution (with 30% concentration) was 500 μL, which contains 22.75 mg of p-coumaric acid].

The results are presented in Figure 14. It was found that the encapsulation efficiency of p-coumaric acid decreased with an increase in the OSA quantity within the hydrogel matrix, which means that the encapsulation efficiency decreased when the cross-linking degree was higher. With the increase in the molar ratio in favor of the –CHO groups, and thus of the cross-linking degree, the polymer network meshes become smaller and cannot immobilize a large amount of Pro. However, a higher Pro amount could be immobilized at a lower cross-linking degree.

### 3.11. Release Kinetics of Pro from Hydrogel Films

**Figure 15:**
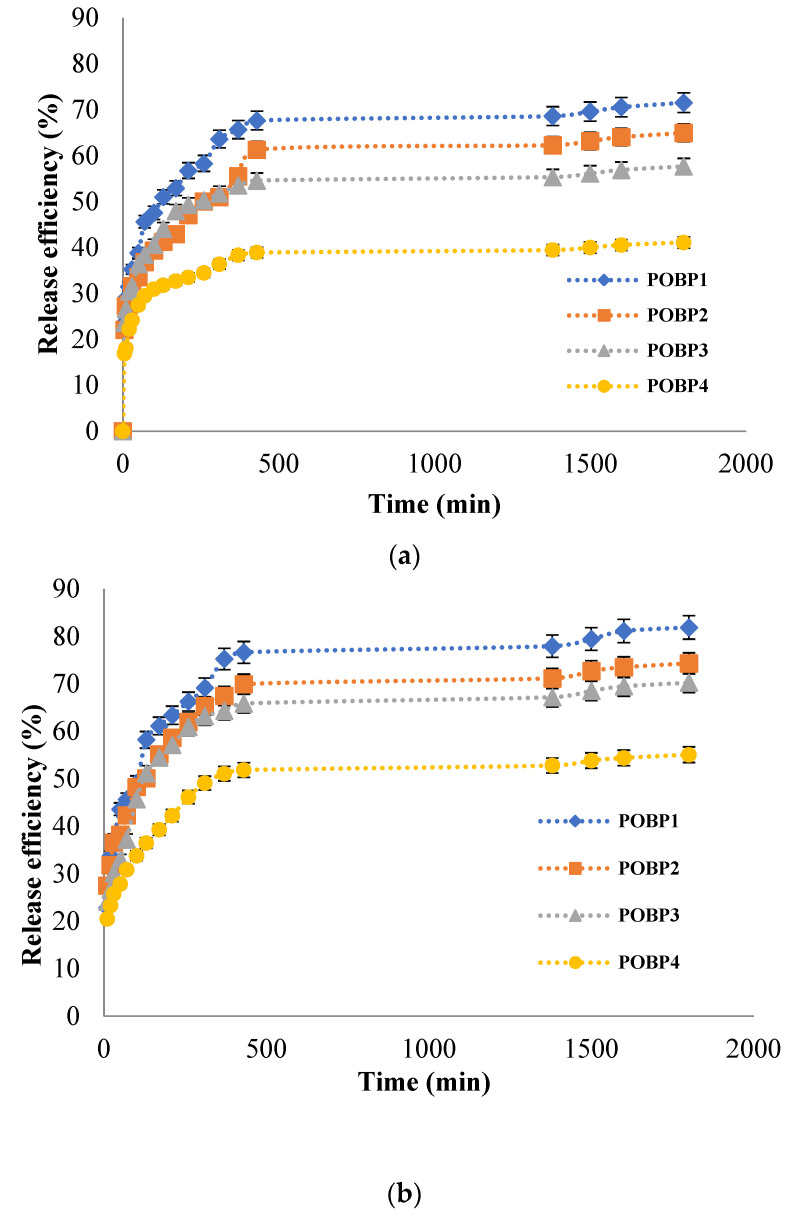

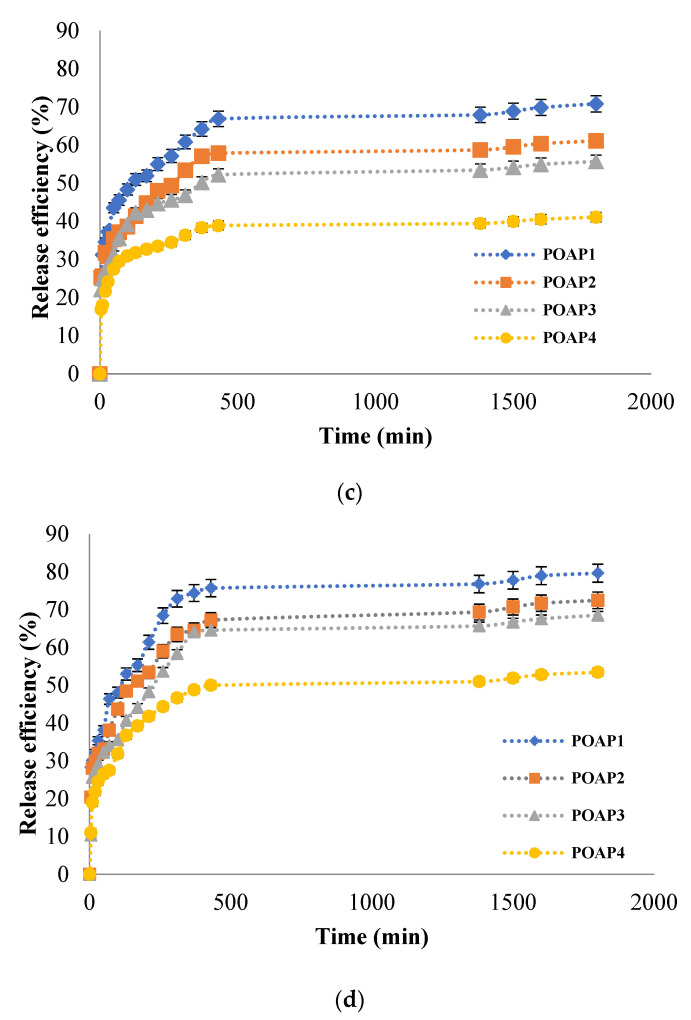
The p-coumaric acid release kinetics in time at different –CHO/–NH_2_ molar ratios of (**a**) hydrogels prepared at pH = 3.5 immersed in acetate buffer solution pH = 5.5; (**b**) hydrogels prepared at pH = 3.5 immersed in phosphate-buffer solution pH = 7.4; (**c**) hydrogels prepared at pH = 5.5 immersed in acetate buffer solution pH = 5.5; and (**d**) hydrogels prepared at pH = 5.5 immersed in phosphate buffer solution pH = 7.4.

The release kinetic studies were performed in vitro in two different pH mediums (pH = 7.4, PBS 0.1 M and pH = 5.5, ABS. 0.1 M using Tween 80 at 1% *w*/*w* concentration) at 37°C for 30 h until equilibrium. Para-coumaric acid was only dosed to study the release efficiency of Pro from the hydrogels; the results obtained are presented in Figure 15.

A controlled release is achieved when the active compound can diffuse out of the film’s network gradually in a limited time with appropriate dosage. Hence, drug waste is avoided, and a suitable treatment effect is reached.

The release of the bioactive principle was rapid in the first phase of the study due to the higher concentration gradient of the drug present at the start of the test, which could act as a driving force for the drug release from the OSA/Gel hydrogels network. The release rate decreases over time until equilibrium; this decrease might be caused by the thickness of the film that acts as a diffusion barrier [13,92,93]. These results are due to the hydrogel’s dense matrix, where strong physical interactions between drug and hydrogel are present, thus limiting drug release from the hydrogel. A research paper by Kapare et al. [97] showed similar results for the encapsulation of propolis on polyvinyl alcohol (PVA)-based hydrogel. 

It was reported that the drug release depends on the interaction between the polymer network and the drug, the hydrogel swelling behavior in an aqueous solution, and the solubility of the drug [74,75]. The OSA/Gel-based hydrogels could be used in biomedical applications for controlled and sustained release of the bioactive principle. It was found that the release efficiency was higher in PBS 0.1 M at pH = 7.4, compared with the release efficiency values obtained in ABS. 0.1 M at pH = 5.5. The maximum p-coumaric acid release efficiency was 82% in PBS at pH = 7.4 after 1800 min; when it was used, the hydrogel synthesized at pH = 3.5 with immobilized Pro (molar ratio –CHO/–NH_2_ was 0.5/1). Furthermore, the p-coumaric acid release efficiency values increased when the cross-linking density (or molar ratio) of the OSA/Gel-based hydrogel was lower. The Pro (p-coumaric acid) release kinetics from OSA/Gel-based hydrogels are consistent with the degree of hydrogel swelling. The results show that the hydrogels with a smaller –CHO/–NH_2_ molar ratio had a higher swelling degree and released a higher p-coumaric acid amount in both pH mediums. These results confirmed that the release efficiency increased with a decrease in the cross-linking degree due to the influence of the polymer network mesh size, which was higher in hydrogels with a lower cross-linking degree. An identical effect has been observed in many other studies. Sarmah et al. Yan et al. and Gull et al. [98,99,100] suggest that increased cross-linking density in hydrogel structures impedes drug release. The tight cross-linking network limits the movement of drug molecules, slowing the release rate or reducing drug release efficiency. Thus, a high degree of cross-linking limits drug diffusion from the hydrogel matrix [98,99,100].

### 3.12. Antioxidant Activity

DPPH possesses an unpaired electron capable of accepting either electrons or hydrogen ions, leading to a color reaction. This characteristic makes it a straightforward and ideal model for assessing free radical scavenging activity. In the scope of this investigation, we assessed the DPPH free radical scavenging activity of OSA/G Pro-encapsulated hydrogels, OSA/G Pro-encapsulated UV-irradiated hydrogels, free Pro, and UV-irradiated free Pro, and utilized ascorbic acid as a standard. The assessment involved determining the inhibition percentage of free radicals from DPPH, and based on these findings, the antioxidant activity was expressed through IC50. The examination of antioxidant activity focused on the immobilized Pro within hydrogel films, allowing for an exploration of the impact of constituent polymers and the molar ratio between polymers on this crucial feature of the active principle.

**Figure 16:**
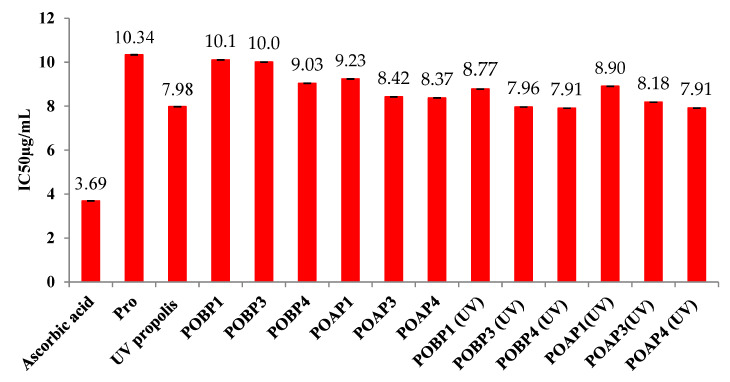
The determined IC50 values for the samples examined for assessing antioxidant activity.

Additionally, the study considered the influence of UV irradiation on the hydrogels and Pro as an active principle. In essence, a lower IC50 value signifies higher antioxidant activity. As mentioned earlier, the IC50 value was calculated from the graphical representation depicting the inhibition percentage versus concentration, expressed in µg/mL. The findings from these analyses are presented in Figure 16.

UV irradiation can significantly affect the antioxidant activity of propolis. Studies show that exposure to UV light can alter propolis’s free radical scavenging capacity, either enhancing or reducing its effectiveness, depending on the preparation and form of the propolis. For instance, a study found that UV irradiation decreased the antioxidant activity of propolis drops, whereas propolis spray showed an increase in antioxidant activity after UV exposure. This difference may be due to the formulation and how each reacts with UV-induced oxidative stress [101,102].

Furthermore, another study demonstrated that propolis methanolic extracts exhibited strong antioxidant activity and provided protection against UV-induced oxidative damage in skin cells, suggesting a potential photoprotective role [102].

These findings suggest that while UV irradiation can sometimes diminish the antioxidant potential of propolis, specific formulations may benefit from UV exposure in terms of increased activity, which can be explored for applications like skin protection against UV damage.

The investigation revealed that the IC50 values for both free Pro and OSA/G Pro-encapsulated hydrogels decrease with an increased amount in the films. This trend was observed for both UV-irradiated and non-irradiated samples and under both pH mediums. Notably, at a pH medium of 5.5, the IC50 value was lower than at a pH of 3.5, suggesting that the antioxidant activity of Pro-loaded hydrogel films intensifies with a higher degree of cross-linking in the hydrogel film. Another noteworthy observation is that the IC50 value decreases upon exposing the samples to UV irradiation. UV irradiation of Pro results in a lower IC50 value than free Pro, indicating enhanced antioxidant activity. This effect is also observed in UV-irradiated, encapsulated propolis, suggesting that UV exposure boosts the antioxidant activity of both free and encapsulated forms. This increase in activity is likely due to the degradation of polyphenols under UV light, which produces lower molecular weight products. Some studies have shown that UV irradiation can increase the antioxidant activity of propolis, particularly in certain formulations like propolis sprays. This enhancement is likely due to the alteration of phenolic compounds, such as flavonoids and phenolic acids, which become more reactive after UV exposure. UV light might also activate latent antioxidants in propolis, making them more effective at scavenging free radicals. Additionally, synergistic effects between modified and existing antioxidants in propolis could further boost its antioxidant potential. However, the impact of UV irradiation is not uniform across all propolis forms, as drops, for example, have been observed to experience a decrease in activity after UV exposure [101,102].

A compelling correlation emerged from the results, revealing that antioxidant content increases concomitantly with a rise in Schiff base cross-linking. Antioxidants inherent in Pro are crucial in neutralizing harmful free radicals, exerting their protective effects.

The observed increase in antioxidant activity in both Pro and Pro encapsulated in hydrogel films following UV irradiation can be attributed to a complex interplay of factors. Propolis contains several antioxidant compounds, including phenolics and prenylates such as flavonoids, p-coumaric acid, ferulic acid, caffeic acid, and drupanine [43,103]; it has inherent properties that contribute to its ability to neutralize free radicals and counter oxidative stress, as highlighted by Andritoiu et al. [43]. UV irradiation acts as a catalyst in this phenomenon, inducing changes in the chemical composition and properties of the natural substances within Pro. This process can either form new compounds or modify existing ones. UV light has been recognized for its capacity to activate phytochemicals by breaking chemical bonds or initiating specific reactions. Such activation can result in the scavenging of free radicals, thereby bolstering the protective capabilities of Pro against oxidative stress and consequently enhancing its overall antioxidant properties [104].

Moreover, UV irradiation has been shown to generate reactive oxygen species (ROS) within Pro. While ROS are commonly associated with oxidative damage, at lower levels, they can paradoxically stimulate the production of endogenous antioxidants within organisms. In the context of Pro exposed to UV light, this phenomenon may trigger the synthesis of its own antioxidants as a defense mechanism [105]. Ebrahimi et al. [106] have demonstrated that exposure of chlorophylls to UV-A light can lead to the generation of singlet oxygen (a reactive oxygen species). Singlet oxygen can oxidize neighboring molecules, and the oxidative stress caused by oxygen triggers a series of biochemical events within the extract, ultimately leading to the synthesis and accumulation of secondary metabolites, including phenolic compounds.

Consequently, this intrinsic response contributes to an overall augmentation of antioxidant activity. This dual action of UV light, activating existing phytochemicals and stimulating the synthesis of endogenous antioxidants, creates a synergistic effect that reinforces the antioxidant potential of Pro. This phenomenon is confined to Pro alone and extends to hydrogel films encapsulating propolis, highlighting the adaptability and responsiveness of this composite system to UV irradiation. These findings underscore the intricate relationship between UV exposure, the chemical dynamics of Pro, and the resulting enhancement of its antioxidant properties, offering valuable insights into potential applications in biomedical and therapeutic contexts.

Indeed, the closer the antioxidant activity of the propolis extracted from the films is to that of unirradiated free propolis, the more effectively the polymer matrix protects the encapsulated propolis. This demonstrates the protective role of the hydrogel film, which helps preserve propolis’ integrity and functionality when exposed to UV light. In medical applications, this protective mechanism is crucial for maintaining the therapeutic efficacy of propolis in various formulations.

## 4. Conclusions

This study successfully developed biocompatible hydrogels based on gelatin by cross-linking protein-free amino groups with aldehyde groups obtained through sodium alginate oxidation. The hydrogels were synthesized with different molar ratios between the oxidized alginate and gelatin, and the effects of pH and cross-linking degree were investigated. The presence of aldehyde groups in the oxidized alginate was confirmed through FTIR and NMR spectroscopy.

The results showed that increasing the molar ratio of NH_2_/CHO led to higher conversion index values of amino groups of gelatin and improved hydrogel stability. However, it also resulted in decreased swelling degree values in different pH mediums. The encapsulation efficiency of propolis increased when the concentrations of oxidized alginate in the hydrogel decreased, while the release efficiency of immobilized propolis decreased with an increase in the cross-linking degree, being in concordance with the swelling degree. Pro immobilization does not affect its antioxidant activity. The IC 50 values of encapsulated Pro are very close to or lower than that of free Pro. The results show that the antioxidant activity increases if the samples are UV irradiated or have more oxidized alginate within the polymeric matrix. These findings also show that the hydrogel films could be UV irradiated for sterilization and used as wound dressings to treat different skin diseases. These findings provide valuable insights into the design and optimization of hydrogels for propolis immobilization and controlled release applications. The developed hydrogels have the potential for use in various biomedical and pharmaceutical fields, such as drug delivery systems or wound healing applications.

## Figures and Tables

**Table 1:** The experimental program used to obtain covalently cross-linked Gel/OSA-based hydrogel films in ABS pH = 5.5 *.

Samples Codes *	Molar Ratio -CHO/-NH_2_	Number of Moles of -CHO × 10^−4^
POA1 (POB1)	0.5/1	1.69
POA2 (POB2)	0.75/1	2.535
POA3 (POB3)	1/1	3.38
POA4 (POB4)	1.5/1	5.07

* The number of moles of –NH_2_ was the same in all experiments, at 3.38 moles/0.2 g Gel. PSB1, PSB2, and PSB3, and PSA1, PSA2, and PSA3, represent the hydrogels prepared by sodium alginate/gelatin using ABS at pH = 3.5 and 5.5, respectively. When preparing hydrogels with SA, we utilized a quantity of polysaccharides equivalent to the oxidized alginate used in creating the hydrogels at various molar ratios, as indicated in Table 1. The prepared hydrogels were coded as 1. POA or POAP (Pro containing hydrogels) when ABS at pH = 5.5 was used; or 2. POB or POBP (Pro containing hydrogels), when ABS at pH = 3.5 was used.

**Table 2:** Thermal characteristics of SA, OSA, Gel, PSA2, POA3 and POAP3.

Samples	Temperature Range (°C)	Weight Loss (%)	Final Residual Fraction (%)	T_max_ (°C)
SA	40–100	13.45	19.18	240
	200–290	37.15		
	558–601	13.8		
OSA	40–100	9.33	28.71	219
	170–300	30.54		
	658–700	14.77		
Gelatin	38–115	7.86	0	310
	230–420	45.68		
	470–660	36.32		
Propolis	40–130	15.38	0	440
	140–300	33.08		
	390–600	38.26		
PSA2	53.18–164	14.01	10.55	250
	180–295	35.24		
	611–680	10.15		
POA3	130–190	11.08	13	240
	200–285	20.35		
	557–610	19.1		
POAP3	131–180	8.21	22.39	237
	200–280	19.99		
	600–690	7.13		

**Table 3:** Conversion index of the samples with Pro.

Samples Codes	Molar Ratio -CHO: NH_2_	Conversion Index (%)
POAP1	0.5:1	63.60
POAP2	0.75:1	72.40
POAP3	1:1	78.24
POAP4	1.5:1	81.78
POBP1	0.5:1	60.79
POBP2	0.75:1	71.93
POBP3	1:1	76.47
POBP4	1.5:1	79.36

**Table 4:** The CI (%) for the hydrogels obtained by chemical cross-linking and the physical interaction between Gel and OSA, respectively, by the interaction of the amine groups of Gel with the carboxylic groups of SA.

Samples	pH of the Medium	Molar Ratio CHO/NH_2_	CI _Chemical Crosslinking +Physical Interaction_ (%)	CI _Physical Interaction_ (%)	CI _Chemical Crosslinking+ Physical Interaction_ − CI _Physical Interaction_ = CI _Chemical Crosslinking (Schiff Base Crosslinking)_ (%)
POB1	3.5	0.5:1	59.31 ± 0.50	17.96 ± 0.38	41.35
POB2	3.5	1:1	75.04 ± 2.10	26.36 ± 0.82	48.67
POB3	3.5	1.5:1	78.53 ± 1.50	28.14 ± 2.15	50.39
POA1	5.5	0.5:1	61.19 ± 3.90	32.15 ± 0.71	34.79
POA2	5.5	1:1	77.98 ± 2.90	37.15 ± 0.40	40.82
POA3	5.5	1.5:1	80.67 ± 0.04	38.57 ± 0.44	42.09
POBP1	3.5	0.5:1	60.70 ± 0.13	20.27 ± 1.70	40.52
POBP2	3.5	1:1	76.47 ± 5.10	28.80 ± 0.97	47.66
POBP3	3.5	1.5:1	79.36 ± 1.03	30.21 ± 2.41	49.15
POAP1	5.5	0.5:1	63.60 ± 1.91	33.21 ± 0.20	30.41
POAP2	5.5	1:1	78.24 ± 0.28	38.69 ± 1.49	39.54
POAP3	5.5	1.5:1	81.78 ± 3.60	40.07 ± 1.04	41.71

## Data Availability

The original contributions presented in the study are included in the article; further inquiries can be directed to the corresponding authors.
